# Stimulation of c-Jun/AP-1-Activity by the Cell Cycle Inhibitor p57^Kip2^

**DOI:** 10.3389/fcell.2021.664609

**Published:** 2021-04-13

**Authors:** Michael Keith Kullmann, Fragka Pegka, Christian Ploner, Ludger Hengst

**Affiliations:** ^1^Institute of Medical Biochemistry, Biocenter, Medical University of Innsbruck, Innsbruck, Austria; ^2^Department of Plastic, Reconstructive and Aesthetic Surgery, Medical University of Innsbruck, Innsbruck, Austria

**Keywords:** p57, CDKN1C, FHL2, AP-1, c-Jun, transcription, HDAC, coactivator

## Abstract

p57 is a member of the Cip/Kip family of cell cycle inhibitors which restrict the eukaryotic cell cycle by binding to and inhibiting cyclin/CDK complexes. They are considered as tumor suppressors and inactivating genomic mutations of p57 are associated with human overgrowth disorders. Increasing evidence suggests that p57 controls additional cellular processes beyond cell cycle control such as apoptosis, cell migration or transcription. Here we report that p57 can stimulate AP-1 promotor activity. While transactivation by c-Jun is strongly activated by p57, it did not enhance c-Fos induced transcription. This indicates that c-Jun is the target of p57 in the canonical AP-1 heterodimeric transcription factor. We could detect endogenous p57/c-Jun containing complexes in cells by co-immunoprecipitation. The strong stimulation of c-Jun activity is not the consequence of activating phosphorylation in the transactivation domain (TAD) of c-Jun, but rather due to negative interference with c-Jun repressors and positive interference with c-Jun activators. In contrast to full-length p57, the amino- and carboxy-terminal domains of p57 are insufficient for a significant activation of c-Jun induced transcription. When expressed in presence of full length p57, the p57 *C*-terminus abrogated and the *N*-terminus enhanced c-Jun activation. This indicates that the *C*-terminus may bind and sequester a putative activator of c-Jun, whereas the *N*-terminus may sequester a c-Jun repressor. Interestingly, the p57 aminoterminus is sufficient for binding to the two c-Jun repressors HDAC1 and HDAC3. These data are consistent with a model of c-Jun activation where p57 is a part of large nuclear remodeling/transcription complexes. p57 might stimulate transcription by inhibiting transcription repressor proteins like HDACs via its *N*-terminus and/or attracting transcription activators through its *C*-terminus. These data suggest that in addition to its role as a CDK inhibitor and tumor suppressor, p57 may also exert tumor promoting functions by activation of the proto-oncoprotein c-Jun.

## Introduction

Mammalian cell proliferation is regulated by proteins of the Cip/Kip family (Sherr and Roberts, 1995, 1999; Hengst and Reed, 1998). The three members p21^Cip1/Waf1^ (p21), p27^Kip1^ (p27), and p57^Kip2^ (p57) share a conserved *N*-terminus which binds to and inhibits the activity of cyclin/CDK complexes. The inhibition of CDK activity restrains cell proliferation and makes all three members of the Cip/Kip family candidate tumor suppressors (Kavanagh and Joseph, 2011). p57 plays an important and unique role in early mammalian development, which was uncovered by the severe developmental defects in p57 knock-out mice (Yan et al., 1997; Zhang et al., 1997). Some of the observed phenotypes resemble many characteristics of Beckwith-Wiedemann syndrome (BWS), a genetic disorder in humans comprising developmental abnormalities and predisposition to tumor development. Of note, mutations in the p57 coding gene CDKN1C can be frequently found in BWS patients and impaired p57 expression or function are most frequent in BWS (Hatada et al., 1996; O’Keefe et al., 1997; Eggermann et al., 2014). Not all of the observed defects in p57 knock-out mice can be explained by impaired cyclin/CDK- and cell cycle regulation (Susaki et al., 2009; Duquesnes et al., 2016). During the last years, novel roles for Cip/Kip proteins in transcription regulation have emerged (Perkins, 2002; Bachs et al., 2018; Creff and Besson, 2020). p57 is known to regulate transcription by direct but also by indirect mechanisms. For example, p57 binds and inactivates CDK7 and CDK9 causing an inhibition of the phosphorylation of RNA Polymerase II *C*-terminal repeat domain (CTD) (Ma et al., 2010) and p57 can also induce the abundance of the myogenic transcription factor MyoD. Either by direct binding to MyoD or by inhibiting cyclin E/CDK2 dependent destabilization of MyoD, p57 was able to promote myogenesis in cell culture models (Reynaud et al., 1999, 2000). Recently, we found that p57 is able to activate gene promoters by interacting with the LIM domain-only protein FHL2 (Kullmann et al., 2020). FHL2 coactivates AP-1 complexes, where the proto-oncoproteins c-Jun and c-Fos form the most active AP-1 heterodimeric complexes (Morlon and Sassone-Corsi, 2003). p57 binds to FHL2 and increases its intrinsic transactivation, probably by competing with inhibitory histone deacetylases (HDACs) for FHL2 binding (Kullmann et al., 2020). AP-1 is composed of homo- or heterodimeric complexes consisting of transcription factors belonging to the basic-leucine zipper (bZIP) family (Hess et al., 2004). AP-1 integrates extracellular signals permitting adaptive cellular changes to alterations in the environment of a cell. Dysregulations in such pathways might lead to increased AP-1 activity and contribute to cell transformation and tumor progression (Jochum et al., 2001; Shaulian, 2010). AP-1 transcription factors bind to palindromic sequences, e.g., c-Jun/c-Fos hetero- and c-Jun/c-Jun homodimers, typically to AP-1 motifs (TPA-responsive elements, TREs), located in the vicinity or longer distances away from the core promoter of regulated genes (Bejjani et al., 2019). One major mechanism of AP-1 stimulation is accomplished by JNK-mediated phosphorylation within the transactivation domain (TAD) of c-Jun, which relieves binding and repression by components of nuclear remodeling complexes (Weiss et al., 2003; Ogawa et al., 2004; Aguilera et al., 2011; Papavassiliou and Musti, 2020). As a consequence of repressor displacement, c-Jun might efficiently interact with the core transcriptional machinery (Weiss et al., 2003; Aguilera et al., 2011) or the composition of AP-1 is shifted from Jun/Jun homodimers to the more stable and transcriptionally active Jun/Fos heterodimers (Ogawa et al., 2004).

Cip/Kip proteins can regulate transcription by CDK-dependent and CDK-independent mechanisms. Among the Cip/Kip proteins, transcription control by p27 is the best studied (Bachs et al., 2018; Razavipour et al., 2020). p27 can act as a transcription repressor in G0/G1-arrested cells and form a complex with several HDACs at promoters of regulated genes (Pippa et al., 2012). In addition, it was found recently that p27 can also positively regulate transcription when phosphorylated at threonine 157 by PI3K effector kinases (Yoon et al., 2019). p27 can form a complex with c-Jun and functions as a coregulator helping to assemble transcriptionally active complexes at AP-1 regulated promoters (Yoon et al., 2019).

When elucidating potential mechanism of FHL2-mediated regulation of AP-1 by p57, we surprisingly observed that following shRNA-mediated knockdown of FHL2, p57 could potently activate an AP-1-dependent reporter gene, indicating that FHL2 might rather block AP-1 activation by p57. These observations are consistent with previous studies which characterized FHL2 also as a corepressor, depending on the cellular context (Martin et al., 2002). We describe that p57 strongly activates transactivation by c-Jun. Our results allow us to propose a model of AP-1 stimulation where p57 forms a complex with c-Jun, which keeps away repressors of c-Jun like HDACs, but simultaneously attracts activators like HATs.

## Materials and Methods

### Plasmids

Oligonucleotide sequences and cloning strategies are presented as Supplementary Data.

Expression vectors pDEST-3xHA-p57, pDEST-3xFLAG-p57, pDEST-3xHA-p57-CK-, pDEST-3xHA-p57-Nt, pDEST-3xHA-p57-Nt-NLS1, pDEST-3xHA-p57-Ct, pDEST-3xHA-p27, and pDEST-3xHA-p21 have been already described (Kullmann et al., 2020). In pDEST-3xHA-p57-CK- the wildtype human p57 sequence codes for a mutant protein (R31A_L33A_W61A_F65A) not binding to Cyclins and CDKs. pDEST-3xHA-p27-Nt expresses an *N*-terminal sequence of human p27 including the kinase inhibitory domain (aa 1–96) in frame with triple HA-tag at the *N*-terminus. The human histone deacetylase 1 and 3 expressing constructs HDAC1/3 FLAG were a gift from Eric Verdin (p plasmid # 13820/13819) and have been described earlier (Emiliani et al., 1998).

pENTR-THT-FHL2 plasmids were obtained by annealing respective pairs of oligonucleotides with *Bgl*II- and *Hin*dIII-restriction site overhangs. Annealing products were ligated into the *Bgl*II-*Hin*dIII sites of pENTR-THT Gateway vector (Sigl et al., 2014). Resulting plasmids were sequence-verified and used in transient transfection experiments or for the cloning of vectors used for lentiviral transduction and conditional knock-down of FHL2. pHR-shFHL2-GFP plasmids were obtained by recombining pENTR-THT-shFHL2 plasmids with pHR-SFFV-DEST-SFFV-eGFP (= pGLTR-FP) (Sigl et al., 2014) and used for the generation of stable cell lines. pSG424-Gal-c-jun expresses a fusion protein of the yeast transcription factor GAL4 DNA binding domain (aa 1–147) fused to human c-Jun (aa 1–223) where DNA-binding and dimerization domains are missing and is described earlier (Hibi et al., 1993). In pSG424-Gal-c-jun4A c-jun sequence was mutated by consecutive overlap extension PCR-mutagenesis (Ho et al., 1989) resulting in 2 serine (aa 63 and 73) and two threonine (aa 91 and 93) exchanges to alanine. See Supplementary Material for sequence information and cloning procedure. pcDNA3-FLAG-Fos WT was a gift from John Blenis (Addgene plasmid # 8966; http://n2t.net/addgene:8966; Addgene_8966) and is described elsewhere (Murphy et al., 2002). pSG424-Gal-c-fos expresses a fusion protein of the yeast transcription factor GAL4 DNA binding domain (aa 1–147) fused to rat c-Fos (aa 213–380) where DNA-binding and dimerization domains are absent and cloned by PCR-amplification of the corresponding codon using pcDNA3-FLAG-Fos WT as a template (oligonucleotide sequences, see Supplementary Material). pFC-MEKK (Agilent) expresses constitutive active MEKK (aa 380–672) which activates JNK.

5×TRE-Luc resembles a synthetic promoter with five consecutive binding sites for AP-1 transcription factor binding sites (“TREs”) in pGL3 basic (Promega), is already described (Diefenbacher et al., 2008) and was kindly provided by Hans van Dam (Leiden). The pFR-Luc reporter plasmid contains a synthetic promoter with five tandem repeats of the yeast GAL4 binding sites in front of a firefly luciferase gene (Agilent). The ubiquitin promoter-driven Renilla luciferase reporter construct pUbi-Rluc controls and normalizes for transfection efficiency.

### Antibodies

Mouse monoclonal FHL2 (F4B2-B11): sc-52667, rabbit polyclonal p57 (C-20): sc-1040, mouse monoclonal p57 (KP39): sc56341, mouse monoclonal anti GAPDH (6C5): sc-32233, c-Jun (G-4): sc-74543, rabbit polyclonal HDAC1 (H-51): sc-7872 and HDAC1 (H-51): sc-7872 were purchased from Santa Cruz, rabbit polyclonal Phospho-c-Jun (Ser73): #9164, rabbit polyclonal Phospho-c-Jun (Ser63) II: #9261 antibodies from Cell Signaling, rabbit polyclonal ANTI-FLAG and mouse monoclonal ANTI-FLAG M2 from Sigma-Aldrich, PSTAIR-motif containing CDKs were detected by using mouse monoclonal anti-PSTAIRE (Yamashita et al., 1992) and mouse monoclonal anti-HA (12CA5 ab16918, Abcam, Cambridge, MA, United States).

### Cell Culture, Transfections and Cell Lysis

The human embryonic kidney (HEK) cell lines 293 and 293T, the human cervix carcinoma cell line HeLa, the human melanoma cell line WM35, the human ductal carcinoma cell line MCF-7, the human osteosarcoma cell line U2OS and the colon carcinoma cell line HRT-18 (also termed HCT-8) were cultured in DMEM (Sigma-Aldrich, St. Louis, MO, United States) supplemented with 10% FBS (PAA) plus 100 U/ml penicillin, 100 μg/ml streptomycin (Sigma-Aldrich, St. Louis, MO, United States) according to ATCC guidelines. CCRF-CEM-C7H2 (CEM) is human acute leukemic cell line glucocorticoid-sensitive subclone of CCRF-CEM (Strasser-Wozak et al., 1995) and HEL is a human erythroleukemia cell line (Martin and Papayannopoulou, 1982; Jäkel et al., 2011). These cell lines were cultured in RPMI-1640 (Sigma-Aldrich). The mouse cardiac muscle cell line HL-1 was cultured in Claycomb media.

A total of 293 and 293T cells were transfected by calcium-phosphate precipitation (Graham and van der Eb, 1973), HRT-18 cell by Polyfect or Lipofectamine 2000 (Thermo Fisher Scientific). Cells were lysed in Laemmli buffer (Laemmli, 1970) or IP-buffer (50 mM Tris pH 7.5, 150 mM NaCl, 0.5% NP-40 and protease inhibitor cocktail (Sigma Aldrich, St Louis, MO, United States) using an ultrasonic homogenizer (Sonoplus, Bandelin, Berlin, Germany).

### Generation of Stable Cell Lines

#### Generation of Stable Gal4-Dependent Luciferase Reporter Gene Cell Lines

The cell line HRT-18FR was generated by transfecting HRT-18 cells with pFR-Luc together with a puromycin-resistance conferring plasmid. Single colonies were isolated under puromycin selection (1 μg/mL) and tested for activation by pSG424-Gal-c-fos. From several positive clones one was selected for low basal and moderate Gal-c-Fos induced reporter activity. The cell line 293FR has been already described (Kullmann et al., 2020).

#### Generation of HRT-18 Cell Lines With Inducible Expression of FHL2 shRNAs

For conditional knockdown of FHL2, sequence verified pENTR-THT-shFHL2 plasmids were recombined with the lentiviral destination plasmid pHR-SFFV-DEST-SFFV-eGFP (pGLTR-FP (Sigl et al., 2014) in a Gateway LR-reaction. The resulting plasmids were verified by restriction digest and lentiviral particle were generated by transient transfection of 293T cells together with the packaging plasmids pSPAX and pMD-G (kindly provided by D. Trono (EPFL, Lausanne, Switzerland). HRT-18 cells constitutively expressing tetR-KRAB, a fusion protein combining the bacterial TetR and the transcription silencing KRAB domain of KOX1 (Deuschle et al., 1995; Sigl et al., 2014) were transduced with lentiviral particles containing FHL2 shRNAs or control luciferase shRNA. The stable pool of HRT-18-shFHL2 cells express respective FHL2 shRNA and eGFP protein in the presence of doxycycline.

### Immunoblotting and Immunoprecipitation

For protein expression and IP experiments cells were harvested 24 to 30 h after transfection and stored as cell pellets at −80°C. Levels of the individual proteins in extracts were determined by Western blotting and if necessary, adjusted before IP. Antibodies for IP were covalently coupled to protein G-agarose beads (Fast Flow 16–266, Millipore). For HA- and FLAG-IPs antibody-coupled beads were incubated with cellular extracts for 4 hours and for IP of p57 over night at 4°C under continuous rotation. After incubation beads were washed at least five times with IP-buffer and proteins eluted from beads by incubation in Laemmli-buffer without DTT at 40°C for 10 min and subjected to immunoblotting. Primary antibodies were detected with horse radish peroxidase-coupled secondary antibodies and enhanced chemiluminescence (ECL).

For endogenous p57-c-Jun CoIP, 1 ml of HRT-18 cell pellet was extracted in 3 ml of lysis buffer (50 mM Hepes pH 7,5, 150 mM NaCl, 1% Triton X-100, 1 mM EDTA, 1 mM EGTA, 10% Glycerol). Protease inhibitors leupeptin (100 μg/ml), aprotinin (100 μg/ml), pepstatin (100 μg/ml), PMSF (10 μg/ml), and 1 mM DTT were added freshly. Following antibodies were used for detection or IP of endogenous p57 from HRT-18 cells: rabbit polyclonal p57 (C-20): sc-1040 and mouse monoclonal p57 (KP39) sc56341, for FLAG-epitope tagged proteins: rabbit polyclonal ANTI-FLAG and mouse monoclonal ANTI-FLAG M2 (Sigma-Aldrich, St. Louis, MO, United States, St. Louis, MO, USA) and HA-epitope tagged proteins: mouse monoclonal anti-HA (12CA5 ab16918, Abcam, Cambridge, MA, United States). Immunoblotting was imaged and processed either by exposing to light-sensitive film followed by developing and fixing or by using the ImageQuant LAS 4000 (Cytivia) machine.

### Luciferase Reporter Gene Assays

HRT-18-sh215 cells were cultured in six well plates in the presence or absence of doxycycline. A total of 2.01 μg DNA including 0.5 μg 5xTRE-Luc reporter, 1.5 μg pDEST, pDEST-3xHA-p57 or pDEST-3xHA-p57-CK together with 10 ng pUbi-Rluc was transfected using Polyfect (Qiagen) according to the manufacturers protocol. A total of 40 h after transfection cells were lysed and luciferase activities determined using the Dual-Luciferase Assay system (Promega, Madison, WI, United States) and a Lumat LB9501 luminometer (Berthold). For quantification firefly luciferase counts were divided by Renilla luciferase counts and expressed relative to control transfected as “fold activation.” Ratio of firefly to renilla luciferase counts was expressed as “relative promoter activity (FL/RL).”

For Gal4-reporter experiments in HRT-18FR cells, six well plate cultures were transfected with a total of 4.03 μg DNA including 20 ng pSG424-Gal-c-jun, 1.5 μg pDEST or pDEST-3xHA-p57 in combination with 2.5 μg pENTR-THT or pENTR-THT-sh215 or pENTR-THT-sh598, and 10 ng Ubi-Rluc using Lipofectamine 2000 (Thermo Fisher Scientific) according to the manufacturers protocol. A total of 72 h after transfection cells were lysed and proceeded as above. For Gal4-reporter experiments in 293FR cells, six well plate cultures were transfected with a total of 2.03 μg DNA including 20 ng pSG424-Gal-c-jun, pSG424-Gal-c-jun4A or pSG424-Gal-c-fos combined with 1.5 μg pDEST, pDEST-3xHA-p57, pDEST-3xHA-p57-CK-, pDEST-3xHA-p57-Nt, pDEST-3xHA-p57-Nt-NLS1, pDEST-3xHA-p57-Ct, pDEST-3xHA-p27 or pDEST-3xHA-p21, and 10 ng Ubi-RLuc using calcium-phosphate precipitation (Graham and van der Eb, 1973). A total of 30 h after transfection cells were lysed and proceeded as above. For titration experiments in 293FR cells, only the composition of the DNA mixture was changed as follows: a total of 2.03 μg DNA included 20 ng pSG424-Gal-c-jun, 0.5 μg pDEST or pDEST-3xHA-p57 combined with 1.5 μg pDEST, pDEST-3xHA-p57-Ct or pDEST-3xHA-p57-Nt, and 10 ng Ubi-RLuc.

### Preparation of Crude Nuclear Extracts

Nuclei from HRT-18 cells were isolated as follows: 10^7^ cells were collected in 1 ml ice-cold lysis buffer (10 mM Tris-HCl, pH 7.5, 10 mM NaCl, 15 mM MgCl2, 250 mM sucrose, 0.5% NP-40, and 0.1 mM EGTA vortexed for 10 sec. and kept on ice for 15 min. Lysate was carefully loaded on top of a 4 ml cold sucrose cushion (30% sucrose, 10 mM Tris-HCl, pH 7.5, 10 mM NaCl, and 3 mM MgCl2) and centrifuged at 1,300 × *g* for 10 min at 4°C. Supernatant was carefully removed and pelleted nuclei resuspended in 1 ml of cold 10 mM Tris-HCl, pH 7.5, containing 10 mM NaCl. After another centrifugation at 1,300 × *g* for 10 min. washed nuclei were extracted in 200 μl extraction buffer (50 mM HEPES, pH 7.5, containing 420 mM NaCl, 0.5 mM EDTA, 0.1 mM EGTA, and 10% glycerol) by sonication. Crude nuclear extract was isolated after centrifugation at 10,000 × *g* for 10 min at 4°C.

### Size Exclusion Chromatography (SEC)

Size exclusion chromatography (SEC) of protein complexes was described before (Grimmler et al., 2007).

Crude nuclear extracts from HRT-18 cells were loaded onto a prepacked Superdex 200 10/300 GL column (GE Healthcare Life Sciences) in 50 mM HEPES, pH 7.5, containing 420 mM NaCl, 0.5 mM EDTA, 0.1 mM EGTA, 10% glycerol and 1 mM DTT. Size exclusion chromatography was performed by collecting fractions (1 ml) at a flow rate of 0.2 ml/min at 4°C using an FPLC/HPLC ÄKTA Purifier (GE Healthcare Life Sciences). Fractions were analyzed using SDS-PAGE and Western blot detection. Molecular weight marker kit MWGF200 (Sigma Aldrich) was used for molecular mass determination and the void volume was determined by using Blue Dextran (Sigma Aldrich).

### Statistical Analysis

Statistical significance was evaluated by the parametric Student’s unpaired two-tailed *t* test using GraphPad Prism version 9.0.1 Values of *p* < 0.05 were considered significant and *p* < 0.01 highly significant. The data are presented as mean ± standard deviation (SD).

## Results

### p57 Activates AP-1 Regulated Promoters in the Absence of FHL2

**FIGURE 1:**
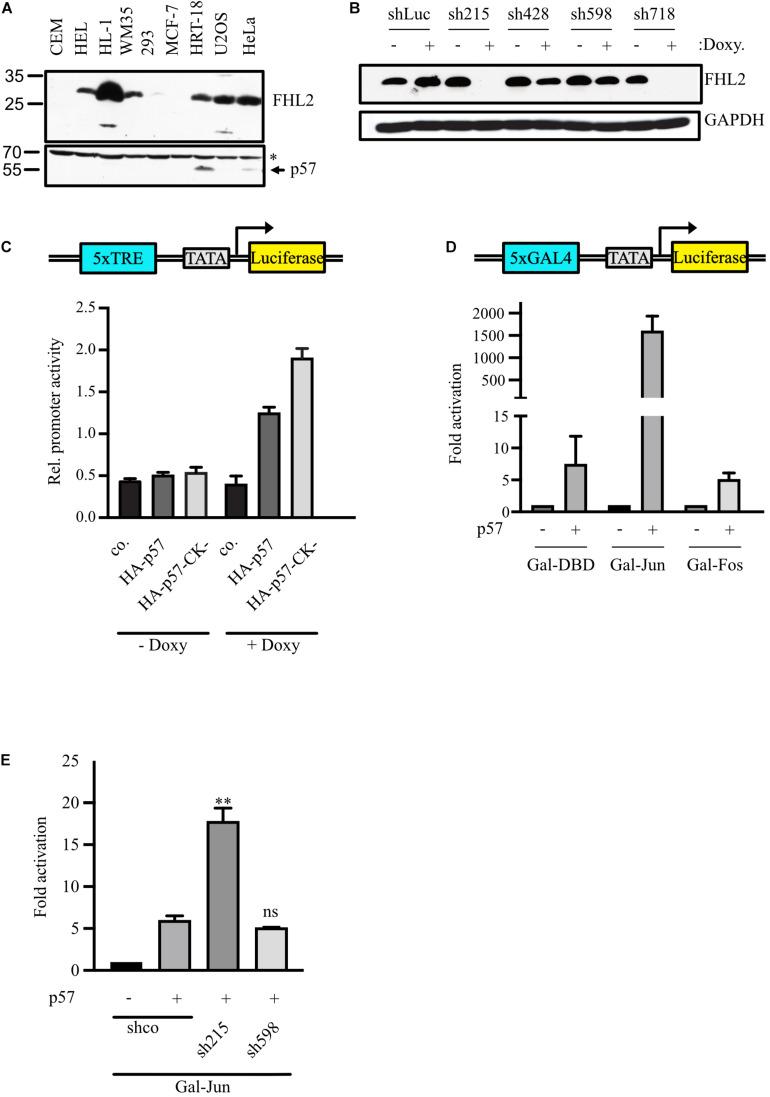
p57 activates AP-1-dependent promoters by inducing c-Jun transactivation independent of FHL2. **(A)** Protein levels of FHL2 and p57 in different cell lines. 100 μg of total protein extract were analyzed by immunoblotting for FHL2 and p57 using a mouse monoclonal FHL2- and a rabbit polyclonal p57-specific antibody. CEM, human leukemic; HEL, human erythroleukemic; HL-1, mouse cardiac muscle; WM35, human melanoma; 293, human embryonic kidney; MCF-7, human breast cancer; HRT-18, human colon cancer; U2OS, human osteosarcoma; HeLa, human cervix carcinoma. The unspecific band at 70 kD (^∗^) after probing for p57 was used to judge equal loading. **(B)** Knockdown of FHL2 using four different doxycyclin-inducible shRNAs in stable pools of lentivirally transduced HRT-18 cells. Western blot analysis of extracts obtained from HRT-18 cells. The expression of shRNAs was induced by the addition of doxycycline (1 μg/mL) for 72 h. Cells expressing shRNA targeting luciferase mRNA (shLuc) served as a control. The FHL2-specific antibody revealed efficient (sh215, sh718) and less efficient (sh428, sh598) inhibition of FHL2 expression. GAPDH served as a loading control. **(C)** Regulation of an artificial AP-1-dependent promotor (schematic representation top panel) by p57. HRT-18.sh215 cells either untreated (– Doxy) or treated with 1 μg/mL doxycycline (+ Doxy) for 72 hours were transfected with 5xTRE-Luc reporter gene construct and 10 ng pUbi-Rluc (expressing renilla luciferase as a transfection control) together with the p57 expression vectors as indicated. A total of 48 h after transfection, cells were harvested and a Dual luciferase assays performed. Relative promoter activities were expressed as firefly luciferase activities normalized for renilla-luciferase activities. Shown are the mean values ± SD from six independent experiments. **(D)** Gal4-dependent luciferase reporter gene experiments comparing the effect of p57 on c-Jun and c-Fos transactivation. 293 cells with stably integrated Gal4-dependent luciferase reporter gene (293FR) were transfected with DNA-expression plasmids for Gal-DBD, Gal-Jun or Gal-Fos and p57. Schematic representation of the reporter construct is shown in the top panel. In addition, 10 ng pUbi-Rluc expressing renilla luciferase were cotransfected as control. A total of 30 h after transfection, cells were extracted and subjected to Dual luciferase assays. Renilla-normalized values are expressed as fold activation relative to control transfected (–) for Gal-Jun and Gal-Fos which are set to 1. For showing lower fold activations, y-axis is split into two segments (bottom: 0 to 15, top: 100 to 2,250). Shown is the mean ± SD from three independent experiments. **(E)** Gal4-dependent luciferase reporter gene experiments analyzing effect of p57 on c-Jun transactivation. HRT-18 cells with stably integrated Gal4-dependent luciferase reporter gene (HRT-18FR) were transfected with DNA-expression plasmids for Gal-Jun, p57 and the indicated shRNAs. pUbi-Rluc (expressing renilla luciferase) was cotranfected as a transfection control. shco was used as a control shRNA not targeting any known mRNA. 72 hours after transfection, cells were harvested and subjected to Dual luciferase assays. Renilla-normalized values are expressed as fold activation relative to control transfected (“–”/“shco”) cells which is set to 1. Shown is the mean ± SD from three independent experiments. Level of significance is indicated. ** = Highly significant, n.s. = not significant.

We recently reported that p57 binds to the transcription cofactor FHL2 and activates FHL2-stimulated AP-1-dependent reporter genes (Kullmann et al., 2020). To elucidate the mechanism of FHL2/AP-1 regulation by p57, we down-regulated FHL2 by small hairpin (sh) RNA. FHL2 protein level vary strongly among different cell lines (Figure 1A). In order to achieve a clear knock-down phenotype for endogenous FHL2 and to be able to study the contribution of p57, we selected the colon carcinoma cell line HRT-18, where FHL2 and p57 are expressed (Figure 1A). We used an inducible system in which the expression of shRNAs can be turned on by doxycycline treatment. Induction of two shRNAs (sh215 and sh718) led to a strong reduction of FHL2 expression, whereas two others (sh428 and sh589) led to a minor reduction of FHL2 expression (Figure 1B). Unexpectedly, the AP-1-dependent reporter construct 5xTRE-Luc, which is activated by p57 overexpression in HeLa cells (Kullmann et al., 2020), was barely induced in HRT-18 cells (Figure 1C). However, upon shRNA-mediated knockdown of FHL2 expression by the addition of doxycycline, p57 coexpression strongly activated the AP-1-dependent reporter (Figure 1C). Interestingly, a p57 mutant which no longer binds and inhibits cyclin/CDK complexes (Kullmann et al., 2020), activated the reporter gene stronger than the wildtype (Figure 1C, HA-p57-CK-), suggesting that activation of AP-1-dependent genes does not rely on the cyclin/CDK binding or inhibition of p57 and that p57 might also inhibit AP-1-activity by a cyclin/CDK-dependent mechanism.

### c-Jun Is the Primary Target of Transactivation Induced by p57

The major AP-1 activity in mammalian cells stems from the heterodimer of the two canonical AP-1 transcription factors c-Jun and c-Fos (Hess et al., 2004). We asked which one of the two factors is regulated by p57. We analyzed the effect of p57 on the transactivation potential of c-Jun and c-Fos in a Gal4-dependent reporter gene system. This Gal4-dependent reporter gene system relies on the activity of a promoter, which consists of five binding sites of the yeast transcription factor Gal4.

We transfected DNA-constructs expressing c-Jun and c-Fos fused to the Gal4-DNA-binding domain (DBD) into 293FR cells which stably express a Gal4-dependend luciferase reporter and express only minimal amounts of FHL2 (Figure 1A, “293”). Of note, Jun- and Fos-Gal4 fusion proteins do not contain sequences important for DNA-binding and dimerization but include their domains for transactivation. Strikingly, p57 induced the Gal4-dependent reporter gene expression more than 1,000-fold in the presence of Gal-Jun (Figure 1D). The activation of Gal-Fos by p57 was much lower and similar to that of the Gal-DBD control (about five-fold, Figure 1D). This result indicated that c-Jun might be the major target of p57 in AP-1-activation.

### Stimulation of c-Jun Transactivation by p57 Can Be Enhanced by FHL2 Knock-Down

Next, we analyzed the transactivation potential of the AP-1 transcription factor c-Jun in a Gal4-dependent reporter gene system in HRT-18 cells which contain a stably integrated Gal4-dependent luciferase reporter gene (HRT-18FR). We found that expression of p57 enhanced reporter activity, confirming the ability of p57 to stimulate the transactivation of c-Jun. The p57-induced activation of reporter activity was further enhanced in the presence of an shRNA efficiently downregulating FHL2 levels (sh215, Figure 1B), whereas less efficient downregulation of FHL2 by sh598 (Figure 1B) led to a stimulation similar to that of the control shRNA (Figure 1E). These data indicate that FHL2 might act as a corepressor for AP-1 in HRT-18 cells and that activation of AP-1-dependent promoters by p57 does not require FHL2. The activation of c-Jun by p57 is most likely the result of enhanced transactivation and not of increased dimerization or DNA-binding of c-Jun, since the Gal-Jun fusion protein lacks the c-Jun DNA binding and dimerization domains.

### c-Jun Is Specifically Activated by p57 Independent of Cell Cycle Inhibition

**FIGURE 2:**
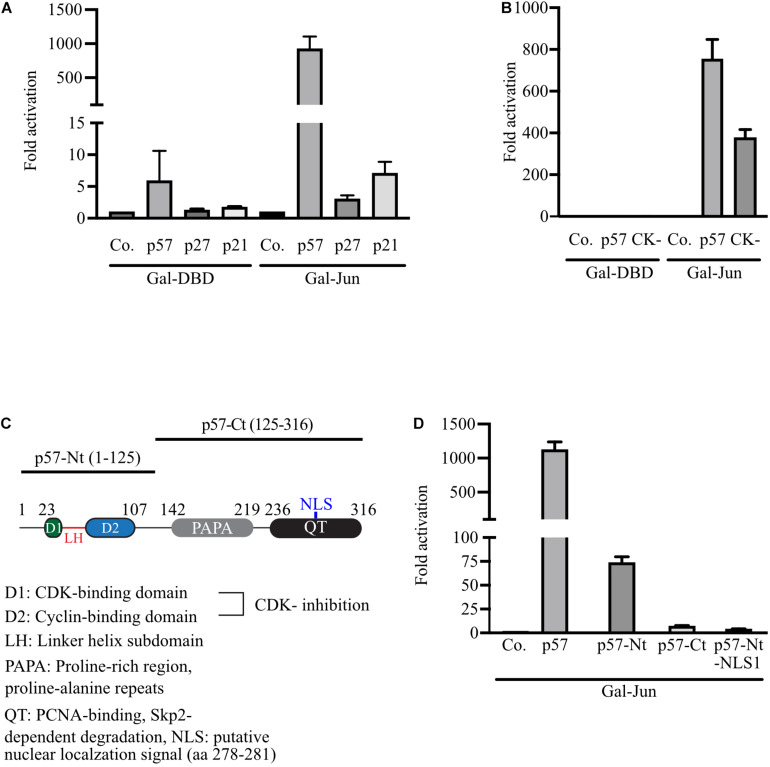
Strong activation of Gal-Jun activity is specific for p57 and requires both, the *N*-terminal and *C*-terminal domains of p57. **(A)** Gal4-dependent luciferase reporter gene experiments comparing the effect of the Cip/Kip-family members p57, p27, and p21 on c-Jun transactivation. 293FR cells were cotransfected with DNA-expression plasmids for Gal-DBD, Gal-Jun and p57, p27 or p21. pUbi-Rluc (expressing renilla luciferase) served as a transfection control. A total of 30 h after transfection cells were harvested and subjected to Dual luciferase assays. Renilla-normalized values are expressed as fold activation relative to control transfected (Co.) which is set to 1. To represent minor activation, the y-axis is split into two segments (bottom: 0 to 15, top: 100 to 1,500). Shown is the mean ± SD from three independent experiments. **(B)** Similar experiment to **(A)** comparing p57 and cyclin/CDK-binding deficient p57 mutant. 293FR cells were cotransfected with DNA-expression plasmids for Gal-DBD, Gal-Jun and p57 or the cyclin/CDK-binding deficient p57-CK- mutant (CK-) and pUbi-Rluc. A total of 30 h after transfection, cells were harvested and subjected to dual luciferase assays. Renilla-normalized values are expressed as fold activation relative to control transfected (Co.) which is set to 1. Shown is the mean ± SD from four independent experiments. **(C)** Schematic representation of p57 and its *N*-terminal and *C*-terminal domains. Key structural or functional regions of human p57 are indicated. Numbers above indicate positions of amino acid flanking distinct regions. Amino acid including the *N*-terminal (p57-Nt) and *C*-terminal (p57-Ct) domains are shown in brackets. **(D)** Experiment as described above **(A)** analyzing the contribution of p57-domains to the activation of Gal-Jun by p57. 293FR cells were cotransfected with DNA-expression plasmids for Gal-Jun and p57 or the indicated p57 domain mutants. A total of 30 hours after transfection cells were harvested and subjected to Dual luciferase assays. Renilla-normalized values are expressed as fold activation relative to control transfected (Co.) which is set to 1. For showing lower fold activations, y-axis is split into two segments (bottom: 0 to 100, top: 100 to 1,500). Shown is the mean ± SD from three independent experiments.

All Cip/Kip family members have been implicated in transcription control. Interestingly, p27 was recently reported to coregulate c-Jun to drive gene expression programs in tumor progression (Yoon et al., 2019). It was observed that the recruitment of c-Jun to many target promoters is p27-dependent and increased by T157 phosphorylation of p27. For p57, we have observed that p57 can act on c-Jun transactivation without the need for DNA binding or dimerization. We therefore aimed to investigate if the regulation of c-Jun by Cip/Kip proteins uses different mechanisms. To compare p27 and p21 with p57 in the regulation of c-Jun-activity in our experimental system, we expressed Gal-Jun together with p57, p27 or p21 and determined Gal4-dependent reporter gene activity in 293FR-cells. Again, we observed a very strong 1,000-fold activation of reporter activity by p57, whereas the effects of p27 and p21 on c-Jun transactivation were neglectable (Figure 2A). This indicates a unique mechanism for p57 in activation of c-Jun and further suggests that activation of c-Jun might not be the consequence of cell cycle arrest induced by all Cip/Kip-proteins. This conclusion is supported by the observation that a p57-mutant lacking cyclin/CDK-binding and -inhibition (p57-CK-) is still able to substantially activate c-Jun in the Gal4-reporter system up to 400-fold (Figure 2B). The drop of activation from 1,000- to 400-fold for p57-CK- might be explained by its incapability to inhibit cyclin/CDKs and arrest the cell cycle. Off note, p21 and p27 were able to activate Gal-Jun by 2- to 5-fold (Figure 2A). It remains to be determined if the reduced ability of the p57-CK-mutant to activate Gal-Jun might also be caused by changes in its subcellular localization or a different affinity of the mutant in Gal-Jun binding or by its inability to bind cyclin/CDK complexes.

### Maximum Activation of c-Jun Requires Full-Length p57

In order to elucidate whether the amino- or carboxyterminal domains of p57 are sufficient for c-Jun-activation, we expressed both domains, p57-Nt comprising amino acid (aa) 1–125 and p57-Ct spanning from aa 126–316 (Figure 2C) in 293FR cells. The activation of coexpressed Gal-Jun was determined in a Gal4-dependend reporter assay. We found that none of the isolated domains activated Gal-Jun comparable to full-length p57 (Figure 2D). Nevertheless, a weak activation by p57-Nt could be detected (Figure 2D). Interestingly, a p57-Nt-mutant which preferably localizes to the nucleus (p57-Nt-NLS1) (Kullmann et al., 2020) was the least efficient p57-mutant in c-Jun-activation (Figure 2D). From these results we concluded that the regions within the *N*- and *C*-terminus of p57 are required for full activation of c-Jun. The small but significant activation by p57-Nt and the clear reduction by its nuclear localized mutant p57-Nt-NLS1 could indicate an additional mechanism of c-Jun activation by p57 acting in the cytoplasm. p57 might add to upstream signaling events, leading to the stimulatory phosphorylation of c-Jun.

### p57-Induced c-Jun Activation Does Not Require c-Jun TAD-Phosphorylation but Is Reduced in the Absence of c-Jun TAD Phosphorylation Sites

c-Jun transcription activity is induced by Jun-kinase (JNK)- mediated phosphorylation of serines and threonines in its TAD (Morton, 2003). JNK1 and JNK2 are phosphorylated and activated by upstream kinases upon various stresses and as a consequence the JNKs shuttle to the nucleus and phosphorylate their target c-Jun (Zeke et al., 2016). Interestingly, p57 was shown previously to regulate JNKs by binding and inhibiting JNK-activity (Chang et al., 2003).

**FIGURE 3:**
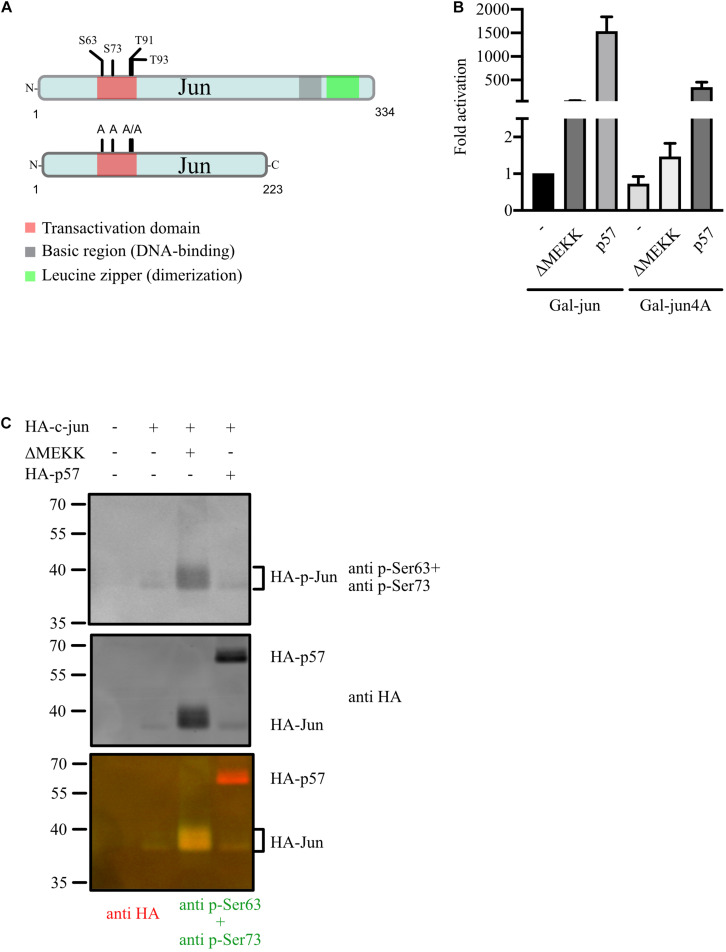
JNK-independent activation of c-Jun by p57. **(A)** Schematic of human wildtype c-Jun, Gal-Jun and Gal-Jun4A mutant. Functional domains and phosphorylation sites in the transactivation domain (TAD) are indicated. A Gal4-DNA binding domain (DBD) fusion in frame to the aminoterminus of c-Jun or mutant c-Jun4A ranging from amino acids 1–223 is used in reporter gene experiments. **(B)** Gal4-dependent luciferase reporter gene experiments analyzing the role of the four major phosphorylation sites in the TAD of c-Jun in the activation by p57. 293FR cells were cotransfected with DNA-expression plasmids for Gal-Jun or the TAD phosphosite mutant Gal-Jun4A in absence or presence of p57 or the constitutive active upstream kinase of JNKs (ΔMEKK). pUbi-Rluc (expressing renilla luciferase) served as a transfection control. A total of 30 h after transfection cells were harvested and subjected to dual luciferase assays. Renilla-normalized values are expressed as fold activation relative to control transfected (Co.) Gal-Jun which was set to 1. For showing lower fold activations, y-axis is split into two segments (bottom: 0 to 2.5, top: 50 to 2,000). Shown are the results from three independent experiments. **(C)** p57 does not induce serine 63 or serine 73 phosphorylation of c-Jun. Immunoblot-analysis investigating the effect of p57 on c-Jun TAD-phosphorylation. 293 cells were transfected with expression vectors for c-Jun and p57 (both HA-tagged) or the constitutive active kinase ΔMEKK. Extracts were prepared 40 h after transfection and subjected to immunoblot-analysis by the LiCor Odyssey system to visualize expression of HA-Jun and Ser 63 and Ser 73 phosphorylation simultaneously. Mixture of Ser 63 and Ser 73 phospho-specific rabbit polyclonal and anti HA mouse monoclonal antibodies were used as first antibodies followed by a mixture of goat derived secondary antibodies coupled with fluorochromes for 680 (anti mouse) or 780 nm (anti rabbit). Three images are shown. Upper: 700 nm channel, phospho-Ser 63/73; middle: 800 nm channel, HA; lower: merged pseudo-colors from channel 700 (red) and 800 (green). Note that molecular weight of HA-Jun is shifted in the presence of the constitutive active kinase ΔMEKK and appears yellow in the merged image.

We tested the regulation of a Gal-Jun mutant by p57 where all major JNK-target serines (S63, S73) and threonines (T91, T93) in the TAD were mutated to alanines (Gal-Jun4A, Figure 3A). The activity of Gal-Jun4A was slightly reduced and barely activated by a constitutive active upstream kinase of JNKs (ΔMEKK) compared to Gal-Jun which confirms the importance of these four phosphorylation sites for c-Jun transactivation. Interestingly, we found that activation of Gal-Jun4A was strongly reduced but still significantly high compared to wildtype c-Jun (Figure 3B). This indicates a strong contribution of c-Jun-phosphorylation in the TAD to the regulation by p57. Therefore, we analyzed the phosphorylation status of serine 63 (S63) and serine 73 (S73) of c-Jun upon p57 expression in transfected 293 cells. We found no change in S63- and S73-phosphorylation by p57 whereas expression of a constitutive active upstream kinase of JNKs (ΔMEKK) strongly induced phospho-signal for S63 and S73 (Figure 3C). From this we conclude that the reduction of c-Jun4A activation by p57 is not the consequence of impaired induction of c-Jun TAD-phosphorylation by p57, but rather due to other mechanisms such as more tight binding of repressor proteins to the c-Jun4A mutant (Weiss et al., 2003). Together, these data indicate that the activation of c-Jun by p57 is not caused by enhanced c-Jun phosphorylation, dimer assembly or DNA binding, but rather by altered recruitment of repressor and activator proteins or altered binding affinities in the presence of p57.

### p57 and c-Jun Are in a Complex *in vivo*

**FIGURE 4:**
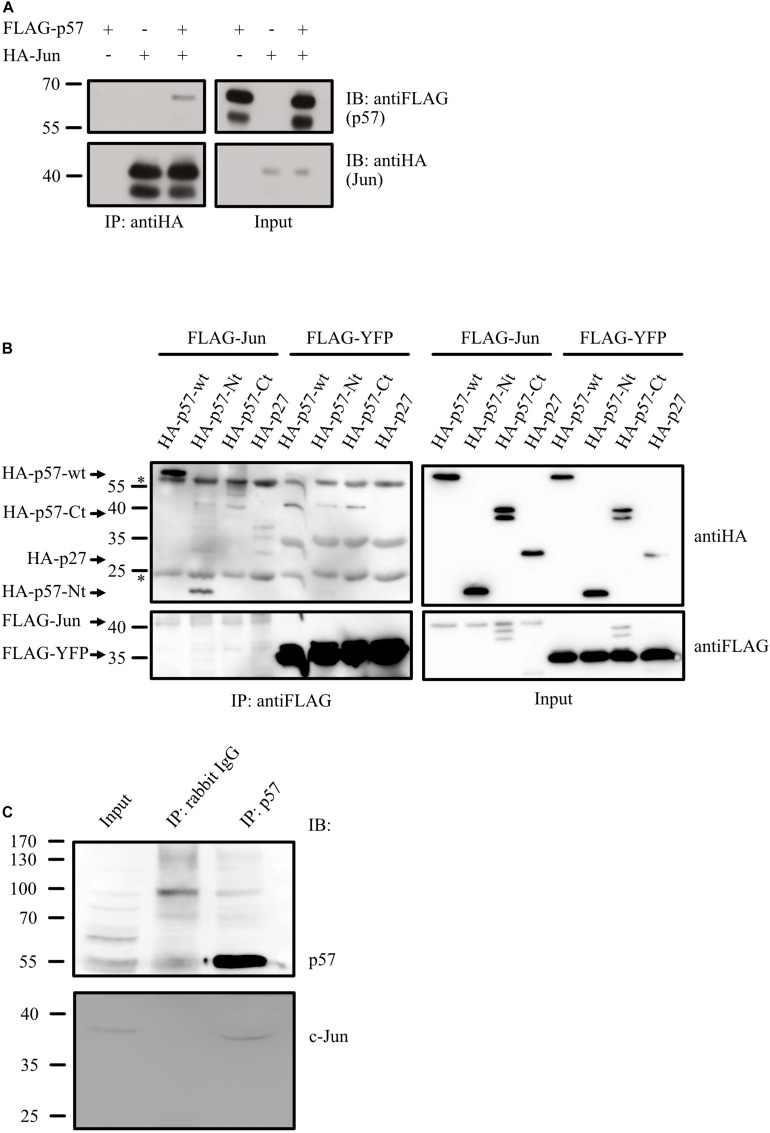
p57 is in a complex with c-Jun. **(A)** Co-immunoprecipitation experiment (Co-IP) followed by immunoblotting showing binding of FLAG-tagged p57 to HA-tagged c-Jun. 293 cells were transfected with expression plasmids for HA-Jun and FLAG-p57. HA-tagged c-Jun was precipitated by using anti-HA mouse monoclonal antibodies. Immunoprecipitates (IP: antiHA) and 1% of the total protein extract used for the IP (Input) were subjected to immunoblot analysis. Upper panels show FLAG-p57, lower panels HA-Jun. Molecular weight markers are indicated left. **(B)** Complex formation of c-Jun with the aminoterminal domain of p57. 293 cells were transfected with expression constructs for FLAG-Jun, HA-p57 and amino- and carboxyterminal domains of p57 (HA-p57-Ct, HA-p57-Nt). FLAG-tagged YFP (FLAG-YFP) was expressed and used as a control. FLAG-tagged c-Jun was precipitated by using anti-FLAG mouse monoclonal antibodies. Immunoprecipitates (IP: antiFLAG) and 1% of the total protein extract used for the IP (Input) were subjected to western-blot analysis. Upper panels show HA-p57 and mutants, lower panels FLAG-Jun/FLAG-YFP. Molecular weight markers are indicated left. Heavy and light chain of the antibodies used in the IPs are indicated (^∗^). **(C)** Endogenous p57 is in a complex with endogenous c-Jun. p57 was immunoprecipitated from extracts of HRT-18 cells using p57-specific rabbit polyclonal antibodies. An IP with rabbit IgG was performed as a control. Immunoprecipitates (IP: p57, IP: rabbit IgG) and 5% of HRT-18 extract used for the IP (Input) were subjected to immunoblot analysis. Upper panel shows p57 and lower panel c-Jun, both detected with mouse monoclonal antibodies. Molecular weight marker is indicated left.

If p57 acts on c-Jun by altering c-Jun coactivator or corepressor complexes, p57 should be present in c-Jun complexes *in vivo*. We first transfected 293T cells with plasmids expressing HA-tagged c-Jun together with FLAG-tagged p57 and found p57 in a complex with c-Jun after HA immunoprecipitation (Figure 4A). Next, we asked whether binding of p57 to c-Jun can be attributed to a specific sequence in p57. Therefore, we expressed HA-tagged p57 and its *N*-terminal and *C*-terminal domains (Figure 2C) together with FLAG-c-Jun and found a significant binding of the aminoterminal domain of p57 (p57-Nt) to c-Jun after FLAG-IP (Figure 4B). Binding of the *C*-terminal fragment (HA-p57-Ct) was not detected. However, because binding of p57-Nt is not that efficient as of p57 wildtype, we cannot exclude that sequences in the *C*-terminal portion of p57 also contribute to c-Jun binding. Interestingly, binding of p57 to c-Jun seems to be more efficient compared to p27 (Figure 4B), a Cip/Kip-inhibitor which was shown before to recruit c-Jun to AP-1 sites in promoters (Yoon et al., 2019). Importantly, binding of p57 to c-Jun does not depend on overexpression, since endogenous c-Jun protein could be detected in immunoprecipitates of endogenous p57 of HRT-18 cells (Figure 4C), supporting the hypothesis that c-Jun and p57 are in a common complex *in vivo*.

### Histone Deacetylases HDAC1 and HDAC3 Bind to the *N*-Terminus of p57

**FIGURE 5:**
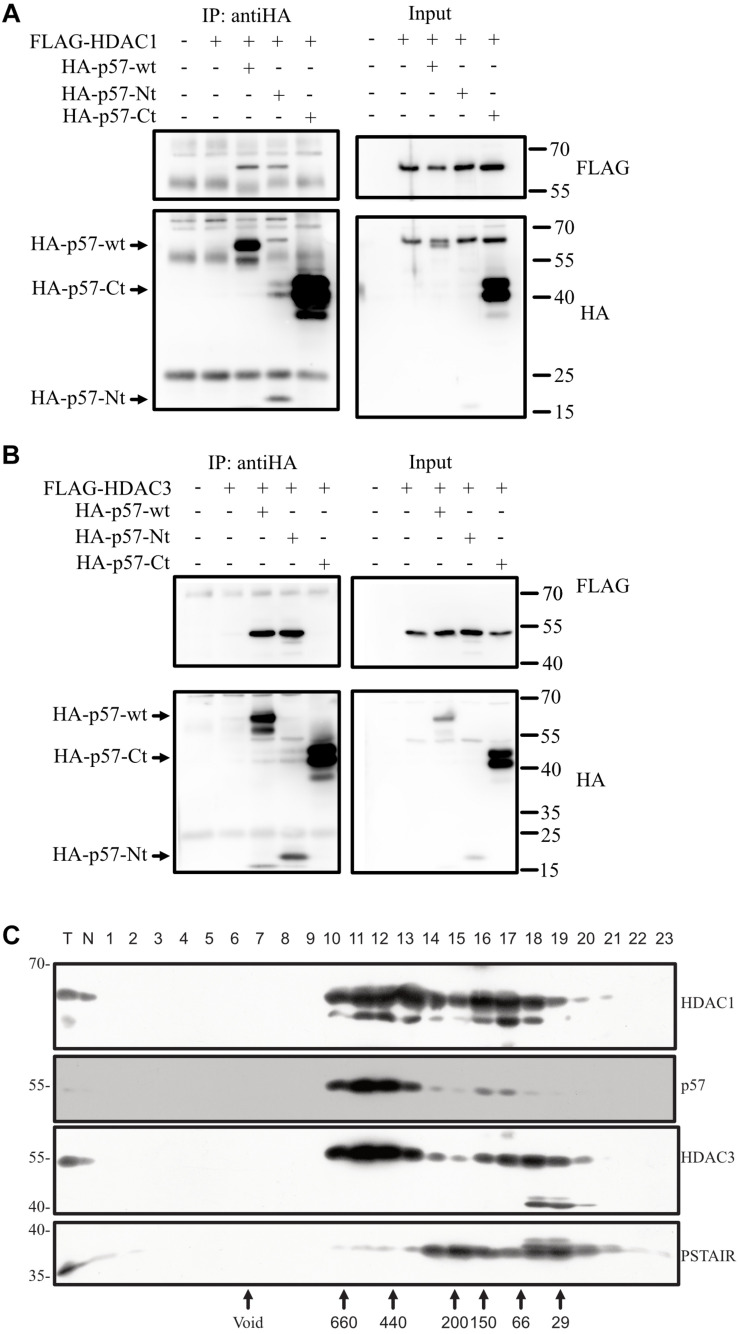
p57 binds HDAC1 and HDAC3 via its *N*-terminal domain and co-migrates in high molecular weight complexes. **(A)** Co-IP followed by immunoblotting investigating binding of exogenous p57 and domains of p57 to HDAC1. HA-p57, aminoterminal (HA-p57-Nt) and carboxyterminal (HA-p57-Ct) domains were expressed in 293 cells together with FLAG-HDAC1. p57 proteins were immunoprecipitated using anti-HA antibodies and the precipitated p57 proteins were detected by using mouse anti-HA antibodies. Coprecipitated HDAC1 was detected with mouse anti-FLAG antibodies (“IP: antiHA”). One percent of protein extract used for immunoprecipitations was loaded onto the same gel and served as an input control (Input). Size of p57 and its domains are indicated by arrows (left) and molecular weight marker is indicated (right). **(B)** Similar experiment as in **(A)** with the exception that binding of FLAG-HDAC3 was analyzed. **(C)** p57 is a component of high molecular weight complexes and coelutes with HDACs. Crude nuclear extracts of HRT-18 cells were applied to size exclusion chromatography. Fractionation by Superdex 200 10/300 GL was followed by SDS-PAGE and Western blotting, using anti-p57 and anti-HDAC1 and HDAC3 antibodies as indicated. Void volume and the elution of molecular weight markers is indicated below. PSTAIR-reactive CDK proteins were detected using monoclonal anti-PSTAIR antibodies.

If p57 is in a complex with c-Jun and thereby regulates c-Jun transactivation, p57 might compete with repressor proteins like HDACs for binding to c-Jun (Weiss et al., 2003). Titrating repressors away from c-Jun might cause induced transcription activity of c-Jun observed in the presence of p57. To investigate this hypothesis, we coexpressed p57 and HDAC1 or HDAC3 and tested for interactions. Both HDACs proteins could be co-immunoprecipitated with p57 (Figures 5A,B), indicating that p57 can form a complex with HDAC1 and HDAC3. We also wanted to determine which part of the p57 protein is required for complex formation with HDACs. We found that HDAC1 and HDAC3 co-immunoprecipitate only with p57-Nt (Figures 5A,B). This indicates that the aminoterminal part of p57 is sufficient for complex formation with HDACs. The interaction of p57 with HDACs might therefore prevent binding to and repression of c-Jun.

### Endogenous p57 of HRT-18 Cells Is a Component of Stable Large Molecular Weight Complexes

Binding of p57 to HDACs in the cotransfection experiments indicates that p57 might enter a high molecular weight complex containing HDACs. To test this, we performed gel-filtration experiments. We fractionated crude nuclear extracts of HRT-18 cells by size exclusion chromatography and detected endogenous p57 in high molecular weight fractions eluting at about 400–700 kD. Only a minor fraction of p57 eluted in fractions of a molecular weight of about 70–150 kD, which could include p57/cyclin/CDK complexes (Figure 5C). Interestingly, HDAC1 and HDAC3 eluted in the same high molecular weight fractions of about 400 to 700 kD, and with a similar pattern as p57. This indicates that p57 might assemble into high molecular weight complexes with so far unknown protein partners, which may contain HDAC1 and HDAC3.

### p57-Carboxyterminus Reduces, Whereas p57-Aminoterminus Enhances c-Jun Activation by p57

**FIGURE 6:**
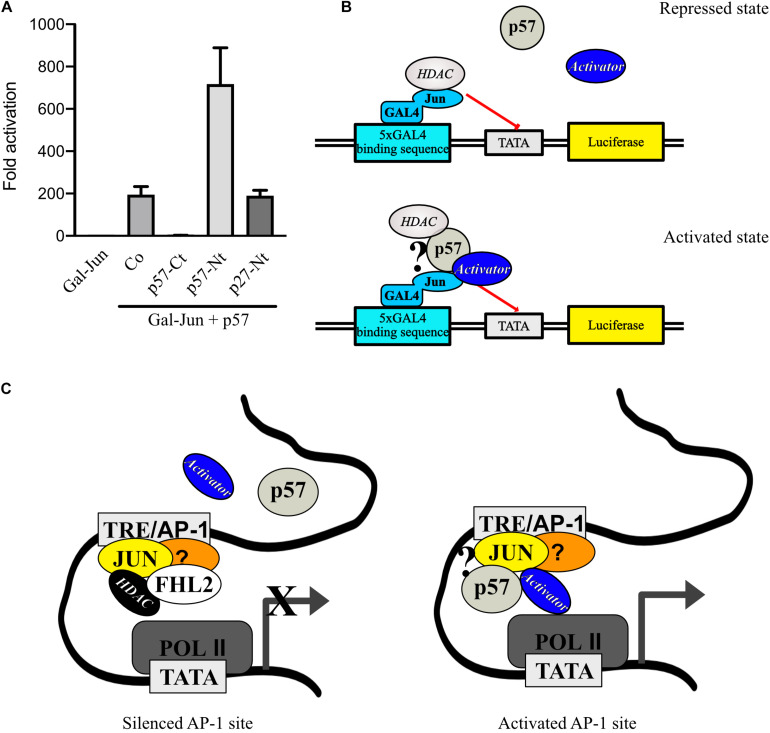
p57 might recruit transcription repressors and activators to c-Jun. **(A)** Gal4-dependent luciferase reporter gene experiments analyzing the effect of p57-domain coexpression on p57-induced c-Jun-activation. Coexpression of p57-Ct may titrate out activators and coexpression of p57-Nt may titrate out repressors of c-Jun activation by p57. 293FR cells were cotransfected with expression plasmids for Gal-Jun, pUbi-Rluc, and p57 wildtype together with vectors expressing p57-Ct or p57-Nt or the related aminoterminal CDK-inhibitory domain of p27 (p27-Nt). A total of 30 h after transfection cells were harvested and subjected to Dual luciferase assays. Renilla-normalized values are expressed as fold activation relative to control transfected (Co.) which is set to 1. Shown is the mean ± SD from three independent experiments. **(B)** Mechanistic models for p57 in stimulating Gal-Jun transactivation function and a schematic of a putative transcription complex. **(C)** Model how p57 and FHL2 might act at endogenous promoters at AP-1 sites (TRE). Question mark in **(B)** and **(C)** indicates that there is no experimental proof for a direct interaction of p57 with the depicted proteins. For details see text.

Although p57-Nt binds HDACs, activation of c-Jun in the Gal4-dependend reporter system was strongly reduced compared to full-length p57 (Figure 2D). This may be due to weaker recruitment of the p57 *N*-terminus into the c-Jun protein complex and/or due to the absence of sequences important for full c-Jun activation. One potential mechanism might be that the missing p57-*C*-terminus contains binding sites for transcriptional activators like histone acetylases (HATs). If so, one would expect that coexpression of the *C*-terminus with p57 wildtype should reduce transactivation of c-Jun by p57 by titrating away a potential activator. Therefore, we cotransfected 293FR cells with full-length p57 and p57-Nt or p57-Ct and tested Gal-Jun activation in Gal-dependent reporter gene experiments. We found a strong reduction of Gal-Jun activation by p57 when p57-Ct was coexpressed (Figure 6A), which supports the hypothesis that a c-Jun-activator binds to the *C*-terminus of p57 and is titrated away when p57-Ct is coexpressed. We also coexpressed the p57 *N*-terminus with p57 wildtype. Based on the immunoprecipitation experiments, we speculated that p57 would preferentially enter c-Jun complexes (Figure 4B). Interestingly, additional expression of the p57 *N*-terminus led to a further activation of Gal-Jun (Figure 6A). This additional activation of Gal-Jun may result from a titration of repressors like HDAC1 and HDAC3 from p57/c-Jun complexes (Figures 5A,B). The aminoterminus of p27 is structurally very similar to the *N*-terminus of p57, and both contain cyclin/CDK binding domains sufficient for CDK inhibition. Interestingly, coexpression of p27-Nt with p57 had no effect on Gal-Jun activation by p57 (Figure 6A). This further supports our earlier conclusion that the mechanisms of AP-1 regulation by p57 and p27 are distinct (Yoon et al., 2019). Taken together, these observations are consistent with a model for the regulation of AP-1 activity by p57, where c-Jun is in a complex with p57. This prevents binding of repressors like HDACs and in addition attracts activators of transcription like HATs leading to a robust activation of AP-1-dependent promoters (Figures 6B,C).

## Discussion

p57 is a potent cell cycle inhibitor and candidate tumor suppressor. The severe developmental phenotype of p57 knock-out mice could be mostly reverted by introducing p27 into the p57 locus revealing the prominent role of the cyclin/CDK-inhibitory function of p57 (Susaki et al., 2009; Duquesnes et al., 2016). However, some phenotypes remained which indicated that p57 might have important non-canonical functions independent of cyclin/CDK-inhibition (Creff and Besson, 2020). One novel non-canonical function of p57 is the regulation of transcription through interaction with transcription factors (Joseph et al., 2009; Creff and Besson, 2020).

We recently reported a positive effect of p57 on the transcription coactivator FHL2 (Kullmann et al., 2020). We found that AP-1-dependent promoters known to be coactivated by FHL2 were stimulated by p57 most likely through induction of the intrinsic transactivation of FHL2 (Kullmann et al., 2020). Here, we report a very strong and FHL2-independent stimulation of AP-1 that depends on the transcription factor c-Jun. Interestingly, we barely detected activation of an AP-1-dependent reporter gene in HRT-18 cells. Surprisingly, upon knock-down of FHL2 however, this reporter became activated and the level of activation corresponded to the level of inhibition of FHL2 expression. This may indicate that at AP-1 sites, FHL2 supports the inhibited state of c-Jun and prevents its activation by p57. Indeed, it has been reported that, dependent on the cell lines used, FHL2 has corepressor or coactivator functions (Martin et al., 2002). This might be caused by the interaction of FHL2 with components of chromatin remodeling complexes like histone deacetylases (Kullmann et al., 2020), which could impose repression on c-Jun transactivation (Weiss et al., 2003). This model would be consistent with the observation that a very strong (1,000-fold) activation of c-Jun-activity by p57 occurs in 293-cells, where only minor levels of FHL2 could be detected.

AP-1 stimulation by p57 required c-Jun, while its most prominent binding partner for heterodimerization c-Fos could not be activated by p57. By analyzing c-Jun and c-Fos proteins which are not able to dimerize or bind to DNA, we could rule out a stimulatory effect on dimerization or DNA-binding by p57. Interestingly, a different mechanism involving c-Jun recruitment to promotors has been recently described for the related CDK inhibitor p27 (Yoon et al., 2019).

The mechanisms controlling AP-1 transcription activity have been extensively studied but are not fully understood yet. One well documented mechanism of potent c-Jun-activation is by phosphorylation of serine and threonine residues in its TAD (Morton, 2003; Papavassiliou and Musti, 2020). Phosphorylation of two critical serine and threonine sites in the TAD of c-Jun overrides binding and repression by corepressor complexes (Weiss et al., 2003; Ogawa et al., 2004; Aguilera et al., 2011). We tested the contribution of c-Jun-phosphorylation in the TAD to c-Jun-activation by p57 and found, while activation by p57 was generally reduced when the 4 phosphorylation sites were mutated to alanine (Jun4A), p57 failed to alter serine 63 or serine 73 phosphorylation. Therefore, one could speculate that the reduced activation of the Jun4A mutant might be due to more tight binding to a component of a repressor complex. Indeed, it has already been reported that a Jun4A mutant can more efficiently titrate out a c-Jun-repressor than the wildtype (Weiss et al., 2003) and also more tightly binds to components of the Mbd3/NuRD repressor complex (Aguilera et al., 2011). Therefore, we tested for HDAC1 and HDAC3 binding to p57 and found both HDACs bound p57 at its *N*-terminus. This could mean that p57 might compete with c-Jun for HDAC-binding thereby enabling c-Jun transactivation function, a mechanism similarly proposed for activation of FHL2 by p57 (Kullmann et al., 2020). However, similar to FHL2, Gal4-Jun was not significantly activated by the aminoterminal domain of p57. This may indicate that the aminoterminus of p57 might only partially relieve c-Jun from repressor inhibition and additional functional domains of the carboxyterminus of p57 might contribute to full activation of c-Jun. The carboxyterminal domain of p57 did not affect c-Jun-activity at all, indicating that it does not bind to c-Jun by itself or does not interfere with components of a putative repressor complex, even though its absence severely impaired the ability of p57 to regulate c-Jun. One model may be that it might bind to activators. To test this hypothesis, we coexpressed p57-Ct with full-length p57 and found a strong reduction of c-Jun-stimulation by p57. This result is in accordance with a molecular mechanism where an activator binds to the p57 carboxyterminus of full-length p57, which is titrated out by the overexpression of the p57-Ct domain that fails to bind to c-Jun (Figure 6B). Interestingly, coexpression of p57-Nt enhanced activation of c-Jun by full-length p57, supporting a model where the *N*-terminus of p57 can bind to HDACs and out-titrate repression of c-Jun (Figure 6B). These observations are consistent with a model for the regulation of AP-1 activity by p57 at promoters where AP-1 activity is silenced by FHL2 (Figure 6C, left panel). Due to its scaffolding function for repressors like HDACs, p57 is blocked from accessing the c-Jun/AP-1 transcriptional complex. By an unknown mechanism, by which FHL2 is removed from such a repressor complex or under conditions of low FHL2 abundance, p57 incorporates by its aminoterminus into a c-Jun/AP-1 transcription complex (Figure 6C, right panel). This prevents binding of repressors like HDACs to c-Jun. In addition, by the carboxyterminus of p57 activators of transcription like HATs are attracted and lead to a robust activation of AP-1-dependent promoters.

We previously demonstrated that ectopically expressed p57 can be found in molecular weight fractions above 600 kD (Kullmann et al., 2020). When analyzing endogenous nuclear p57, we observed that a surprisingly large amount of p57 coelutes with molecular weight fractions of 400–700 kD. It will be interesting to identify p57 associated proteins in such high molecular weight complexes. These may include repressors or stimulators of transcription. Since p57 is an intrinsically disordered protein (IDP; Galea et al., 2008; Huang et al., 2015), p57 could have a scaffolding function in the arrangement and rearrangement of such complexes or might directly regulate the activity of histone modifiers. It remains to be determined if p57 can directly interact with HDAC1 and HDAC3. If so, p57 might modulate their enzymatic activities. However, it was previously described that p57 can directly bind to HDAC7 in a protein interaction screen (Duquesnes et al., 2016). The composition of chromatin remodeling complexes which control AP-1 transcription activity depend on the physiological context (Ogawa et al., 2004; Aguilera et al., 2011), and especially late molecular events leading to full transcription activation of c-Jun/AP-1 by coactivators are not fully understood yet. One such activators are the highly homologous CBP (CREB-binding protein) and p300, which both contain transcription stimulating histone acetylase activity (Goodman and Smolik, 2000; Chan and Thangue, 2001). It was shown that CBP binds to serine 63 and serine 73 phosphorylated c-Jun to stimulate its transactivation (Bannister et al., 1995). Interestingly, it was reported that p21 can stimulate p300 transactivation (Snowden et al., 2000) and p27 can bind together with c-Jun and CBP at promoters of AP-1 regulated genes (Yoon et al., 2019). Our experiments suggest that p57 uses a different mechanism than p21 and p27 for activation of c-Jun. This is emphasized by the observation that in contrast to p57, p21, and p27 could not significantly activate Gal4-Jun in the Gal4-dependent reporter system, where DNA binding or dimerization are not required for transactivation. In our studies we found a potent activation of AP-1/c-Jun-activity through p57 by using reporter gene experiments. Previously, we reported the activation of a MMP-1-Luc construct by p57 (Kullmann et al., 2020). This reporter is controlled by the promoter region −517/+63 from the human MMP-1 collagenase gene (Angel et al., 1987). Whereas this region is not solely dependent on AP-1 transcription factors, the 5xTRE-Luc reporter used in this study strictly depends on AP-1 transcription factors. The molecular composition of AP-1 dimers bound to the 5xTRE sequence could not be determined in these experiments, but most likely c-Jun/c-Jun homo- or c-Jun/c-Fos heterodimers are the dominant factors targeted by p57. By using the Gal4-dependent reporter gene system, we were able to identify c-Jun as the relevant AP-1 transcription factor activated by p57.

So far, these observations are limited by the use of artificial reporter gene systems. We can currently only speculate about the physiological relevance of the results on physiological AP-1-dependent promotors. It will be important to demonstrate next that p57 is in fact associated with AP-1 sites on endogenous promoters to regulate the transcription of respective genes. Systematic approaches using chromatin immunoprecipitation technology (ChIP) may be advantageous over testing single genes known to be regulated by AP-1 transcription factors, because they might reveal a contribution of p57 to specific promoters and genetic programs. One could imagine that re-expression of the paternally silenced p57 gene might contribute to cancerogenesis by acting as a transcriptional co-activator of pro-proliferative factors like AP-1/c-Jun. Furthermore, our observations using the Gal4-reporter system indicated the regulation of transcriptional repressors or activators by p57. Proteomic approaches should enable the identification of p57 in multiprotein transcription complexes. Although this reporter system does not completely reflect the situation at endogenous promoters, it is evident that the molecular details of the strong c-Jun-activation by p57 is worth to be further explored in order to gain knowledge about novel molecular targets and mechanisms of p57 in transcription control.

The biological significance of c-Jun-activation by p57 remains to be investigated. Both, p57 and c-Jun are important for embryonic development (Eferl and Wagner, 2003; Creff and Besson, 2020; Papavassiliou and Musti, 2020). A total of 10% of p57 knockout mice die between E13 and E16 (Yan et al., 1997; Zhang et al., 1997, 57) and c-jun −/− mice die around E13 (Hilberg et al., 1993). c-Jun is important for proper heart and liver development (Eferl et al., 1999) and p57 is expressed from the embryonic stages on until adulthood of mice in the heart (Matsuoka et al., 1995; Yan et al., 1997; Takahashi and Nakayama, 2000). One could imagine that p57 might modulate c-Jun-activity in transcription events during heart development and it would be interesting to investigate a possible collaboration of the two proteins in cardiomyocyte differentiation model cell systems.

p57 is a cell cycle inhibitor and considered as a tumor suppressor (Creff and Besson, 2020) whereas the role of AP-1, in specific c-Jun, is rather pro-proliferative (Eferl and Wagner, 2003). We found that a mutant of p57 which does not inhibit CDKs and cell cycle progression (p57-CK-) can still activate c-Jun. This suggests a cyclin/CDK-independent role of p57 as a coregulator of c-Jun. It may also be that specific modifications stimulate or inhibit the recruitment of p57 in c-Jun complexes. Additional insights to the relevance of c-Jun/AP-1-activation by p57 could be investigated in chromatin immunoprecipitations and identification of regions/binding sites for p57. This may hint to the physiological and pathophysiological processes where p57 transcription regulation could be of importance. It is tempting to speculate that p57, due to its interaction with specific transcription factors like c-Jun or with general components like histone modifiers in larger transcription complexes, might modulate a broader spectrum of transcription programs.

## Data Availability Statement

The raw data supporting the conclusions of this article will be made available by the authors, without undue reservation.

## Author Contributions

FP, CP, and MK designed and carried out the experiments. FP performed p57/c-Jun interaction experiments. CP produced the inducible knock-down cell line. MK conducted the project and wrote the article. LH wrote and revised the manuscript. All authors discussed the results, commented on the manuscript text, and approved the final version submitted.

## Conflict of Interest

The authors declare that the research was conducted in the absence of any commercial or financial relationships that could be construed as a potential conflict of interest.
