# Supplementary material for: Stimulation of c-Jun/AP-1-Activity by the Cell Cycle Inhibitor p57Kip2
Source: Front Cell Dev Biol. 2021 Apr 13;9:664609. doi: 10.3389/fcell.2021.664609 (PMC8076676; doi:10.3389/fcell.2021.664609)

Supplementary Material

1 Supplementary Data

Oligonucleotides for small hairpin RNA expression

shFhl2-215-sense/antisense:

5’-gatccccgaaactcactggtggacaattcaagagattgtccaccagtgagtttctttttggaaa-3'/

5’-agcttttccaaaaagaaactcactggtggacaatctcttgaattgtccaccagtgagtttcggg-3'

shFhl2-428-sense/antisense:

5’-gatccccccaaagacaatcagaatttttcaagagaaaattctgattgtctttggtttttggaaa-3'/

5’-agcttttccaaaaaccaaagacaatcagaattttctcttgaaaaattctgattgtctttggggg-3'

shFhl2-598-sense/antisense:

5’gatccccttcacagctcgcgatgactttttcaagagaaaagtcatcgcgagctgtgaatttttggaaa-3'/

5’-agcttttccaaaaattcacagctcgcgatgacttttctcttgaaaaagtcatcgcgagctgtgaaggg-3'

shFhl2-718-sense/antisense:

5’-gatccccgaggaacggcagtggcatattcaagagatatgccactgccgttcctctttttggaaa-3'/

5’-agcttttccaaaaagaggaacggcagtggcatatctcttgaatatgccactgccgttcctcggg-3'

shLuc (Luciferase-shRNA):

5'-gatcccccttacgctgagtacttcgattcaagagatcgaagtactcagcgtaagtttttggaaa-3'

5'-agcttttccaaaaacttacgctgagtacttcgatctcttgaatcgaagtactcagcgtaagggg-3'

Numbers indicate target sequence in coding region of human FHL2.

Oligonucleotide pairs for shFHL2-215, shFHL2-428, shFHL2-598 and shFHL2-598 were annealed and phosphorylated producing a 5’- and 3’-overhang suitable for ligation into the BglII-HindIII-sites of pENTR-THT.

Oligonucleotides used to clone pSG424-Gal-c-fos

pSG424-Gal-c-fos was cloned by using the primer pairs:

rat c-fos (aa 213-for)+BamHI:

5’-ttttggatcccagaggagatgtctgtgacc-3'

rat c-fos (aa 380-rev)+Acc65I:

5’-ttttggtacctcacagggctagcagtgtgg-3'

in a PCR reaction with pcDNA3-FLAG-Fos WT as a template. PCR-product was BamHI-Acc65I digested and ligated into the BamHI-Acc65I-sites of pSG424.

Oligonucleotides used to clone pSG424-Gal-c-jun4A

c-jun (S73A)-for: 5'-ggggctgctcaagctggcggcgcccgagctggagcgcctg-3'

c-jun (S73A)-rev: 5'-caggcgctccagctcgggcgccgccagcttgagcagcccc-3'

pSG424-rev (40-20): 5'-cctctacaaatgtggtatggc-3'

pSG424-for (3074-3096): 5'-ggaatcaaggctagaaagactgg-3'

c-jun (T91A/T93A)-for: 5'-gggcacatcaccaccgcgccggcccccacccagttcctg-3'

c-jun (T91A/T93A)-rev: 5'-caggaactgggtgggggccggcgcggtggtgatgtgccc-3'

c-jun (S63A)-for: 5'-gacctcctcaccgcgcccgacgtgggg-3'

c-jun (S63A)-rev: 5'-ccccacgtcgggcgcggtgaggaggtc-3'

Template DNA of the first round of overlap-extension(Ho et al., 1989) was pSG424-Gal-c-jun, products from the overlap-extensions were use as templates for the next round of mutagenesis. The last product was XhoI-SacI digested and ligated into the XhoI-SacI sites of pSG424 obtaining pSG424-Ga-c-jun4A.

2 Supplementary Figures

Supplementary figure 1: Original ECL-exposed film originated from two PVDF membranes probed for p57 or FHL2; used for final figure 1A , cropped areas are indicated.

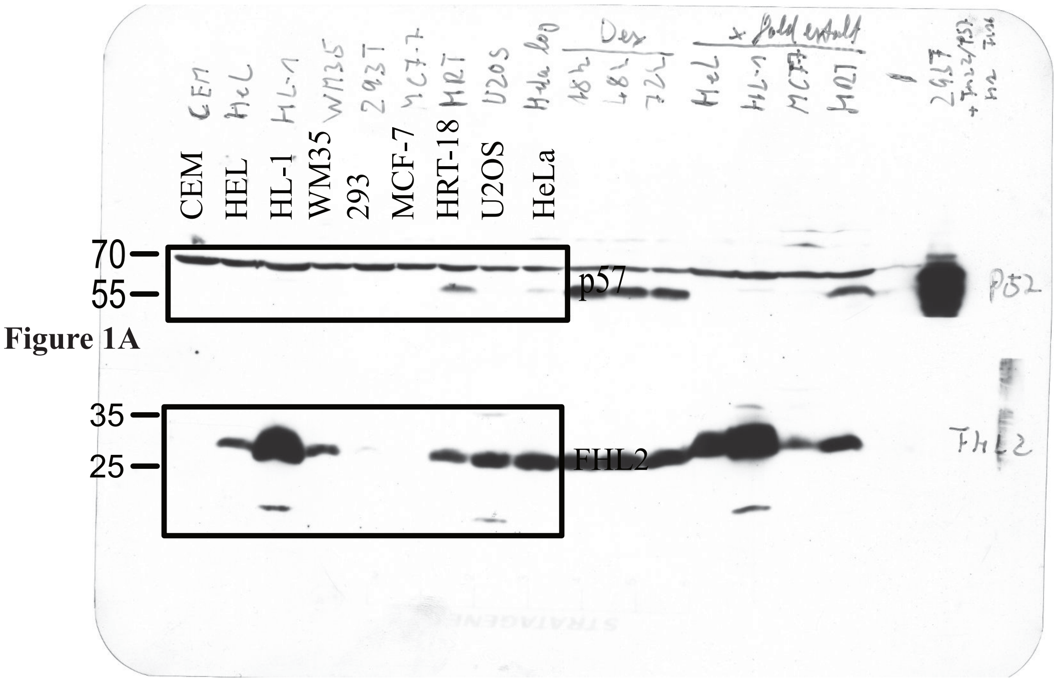

Supplementary figure 2: Original ECL-exposed film (upper) and Odyssey-scan (lower) used for final figure 1B , cropped areas are indicated.

Figure 1B

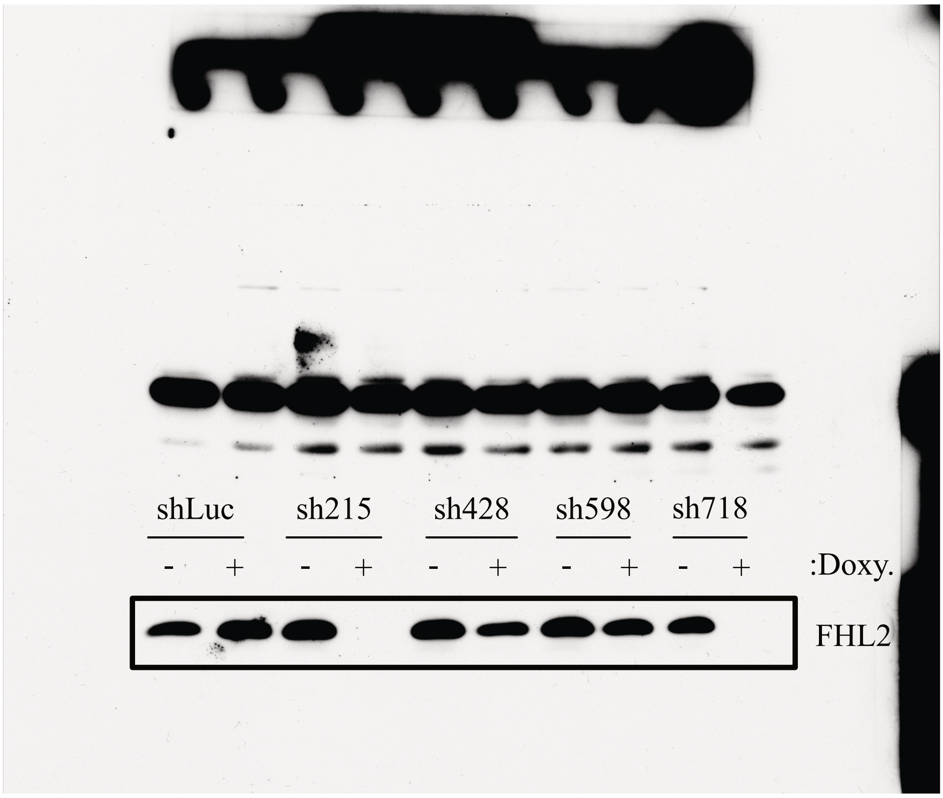

Figure 1B

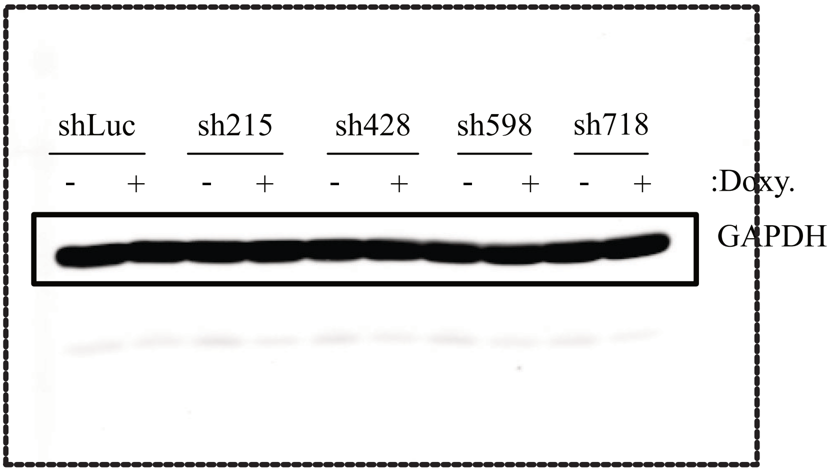

Supplementary figure 3: Odyssey-scans used for final figure 3C, cropped areas are indicated.

Figure 3C

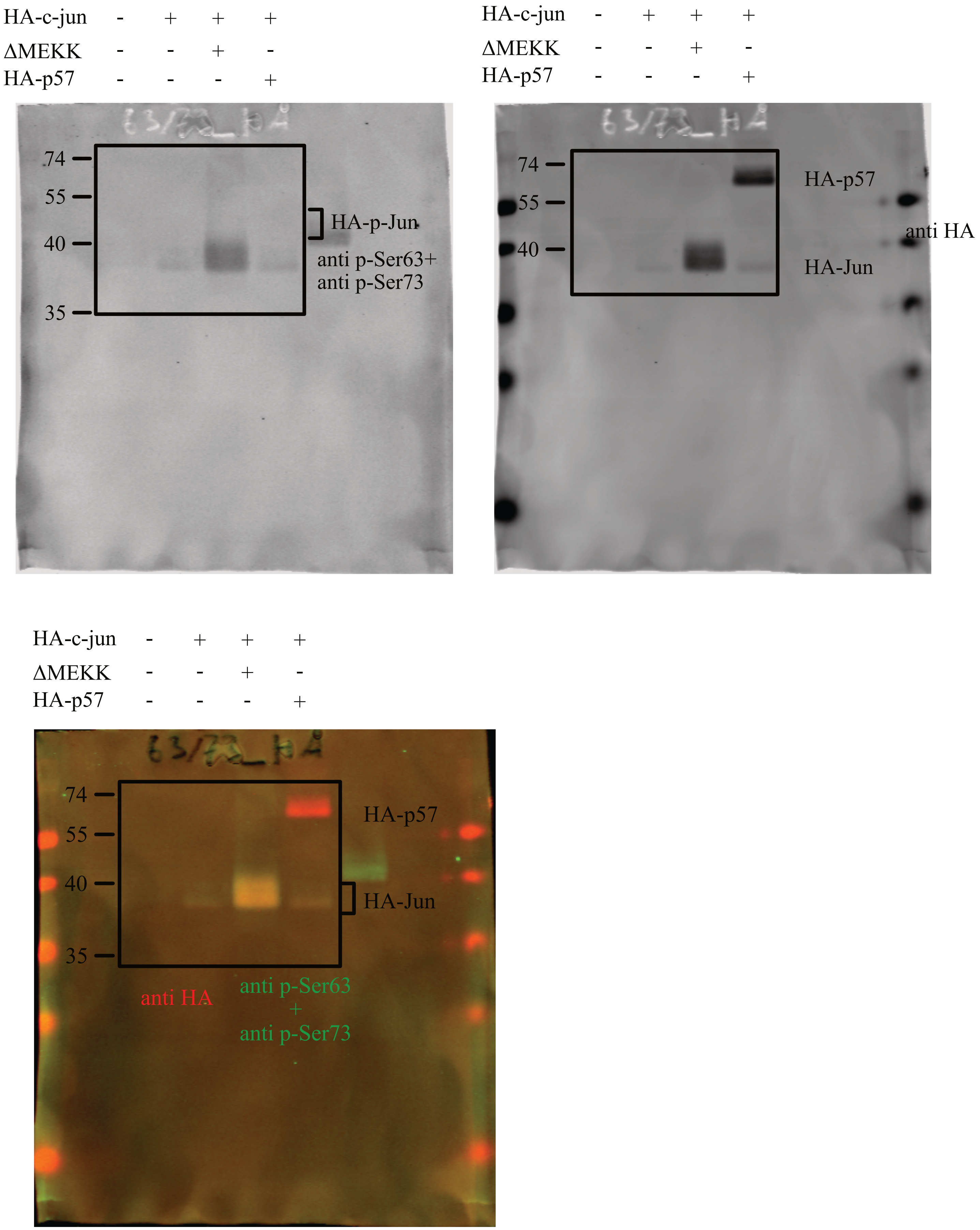

Supplementary figure 4: Original ECL-exposed film (upper) processed for final figure 4A, cropped areas are indicated.

Figure 4A

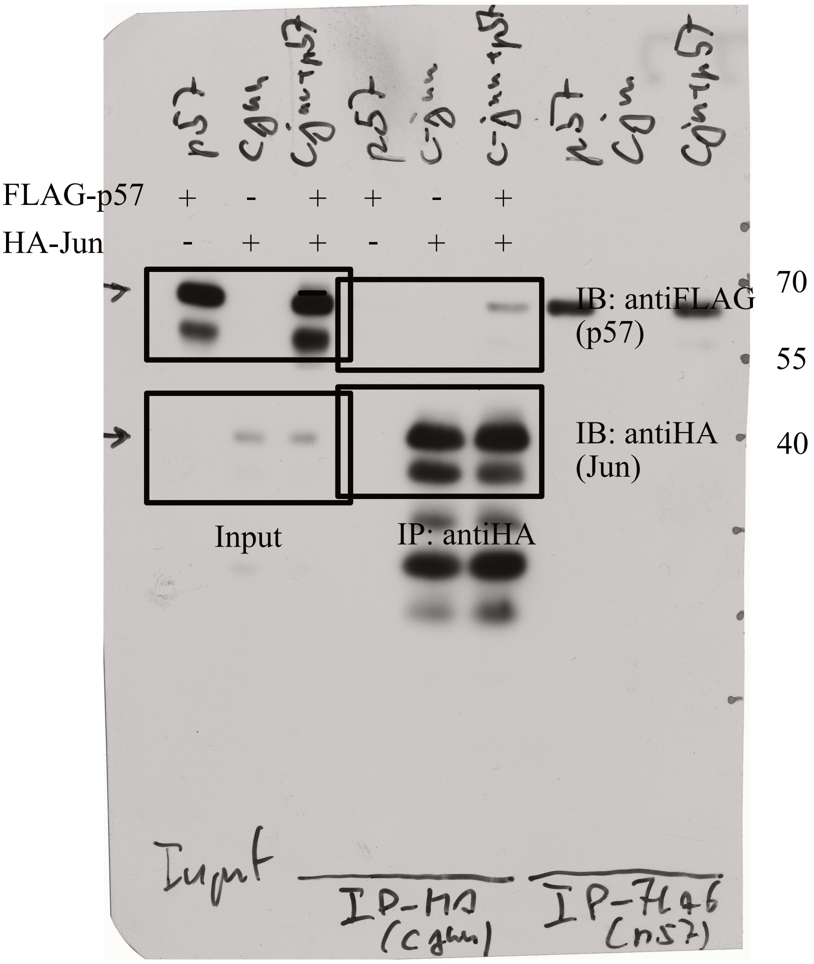

Supplementary figure 5: LAS4000 pictures processed for final figures 4B and 4C, picture borders are indicated by dotted squares and cropped areas are by full lane frames. For figure 4C a shorter exposure, for c-Jun a longer exposure was used for cropping (left and right images).

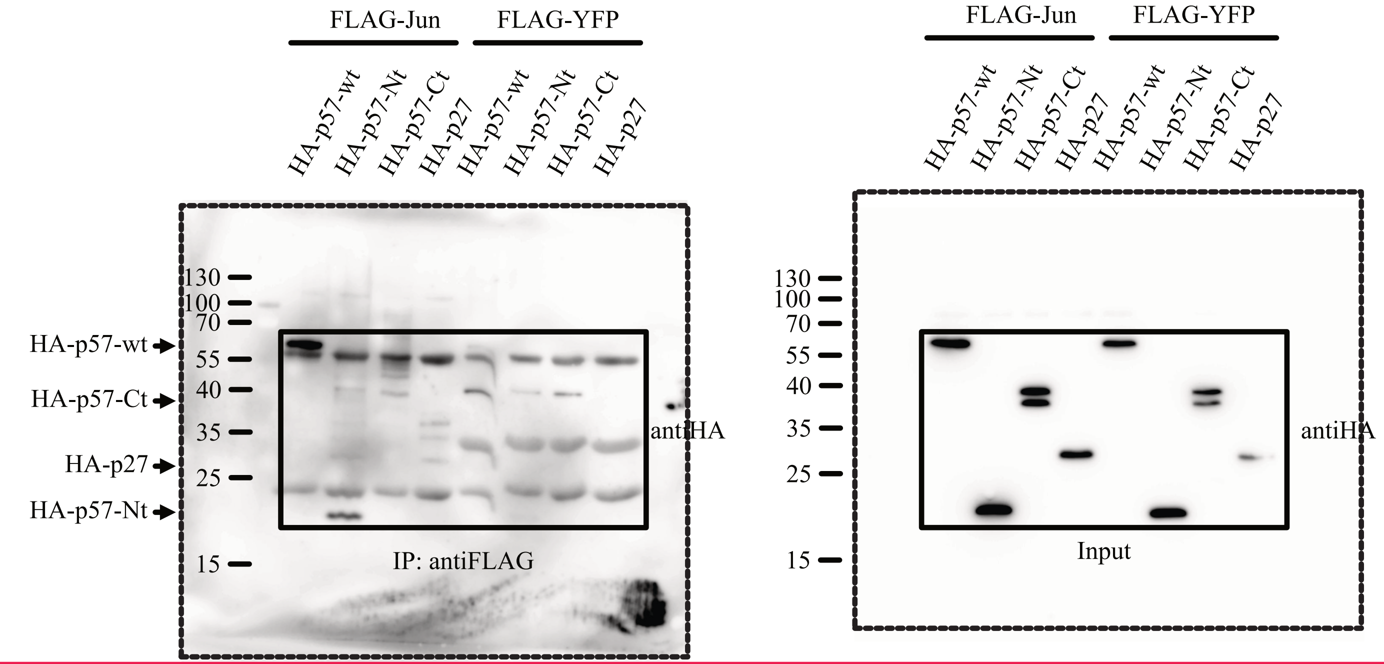

Figure 4B

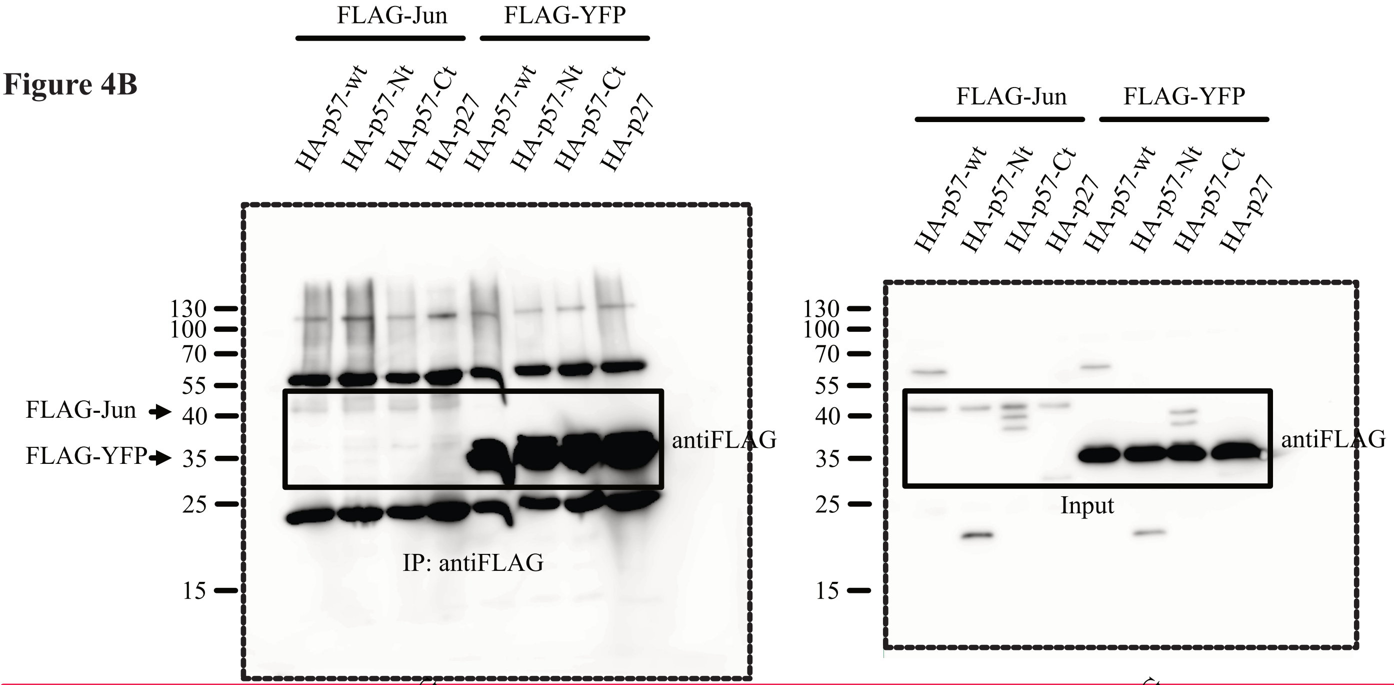

Figure 4C

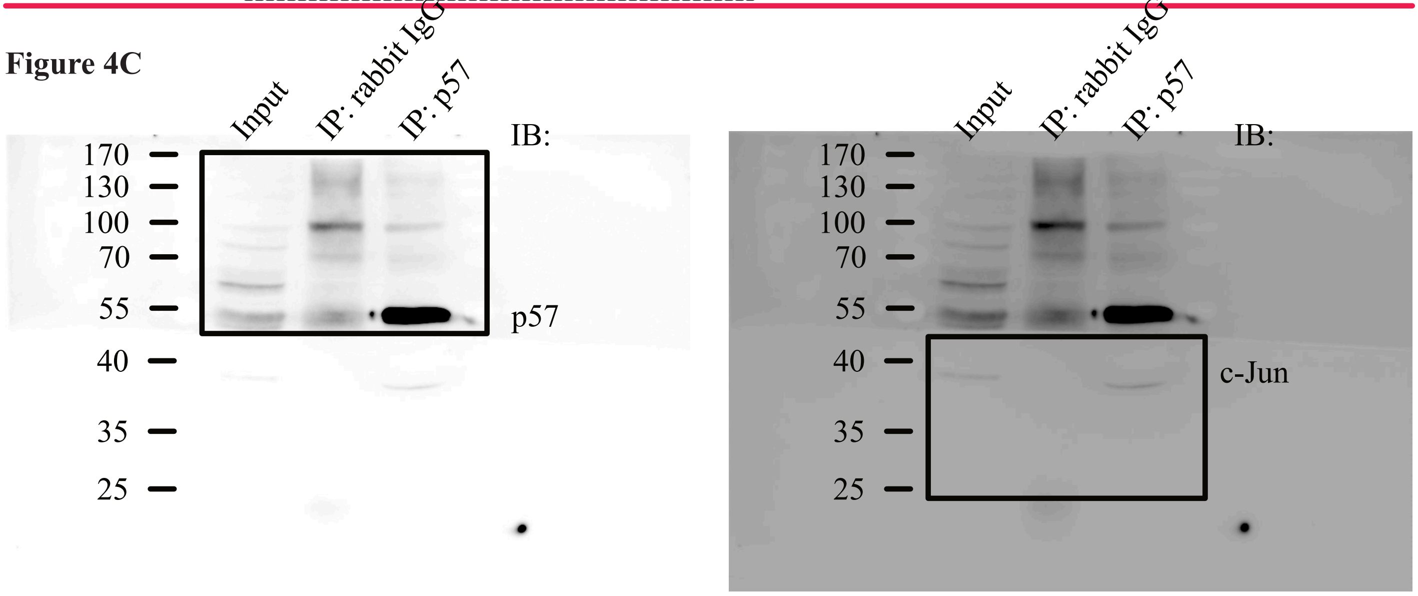

Supplementary figure 6: Original LAS4000 scans cropped for preparing final figure 5A. Cropped areas and boundaries of Las4000 scans are indicated.

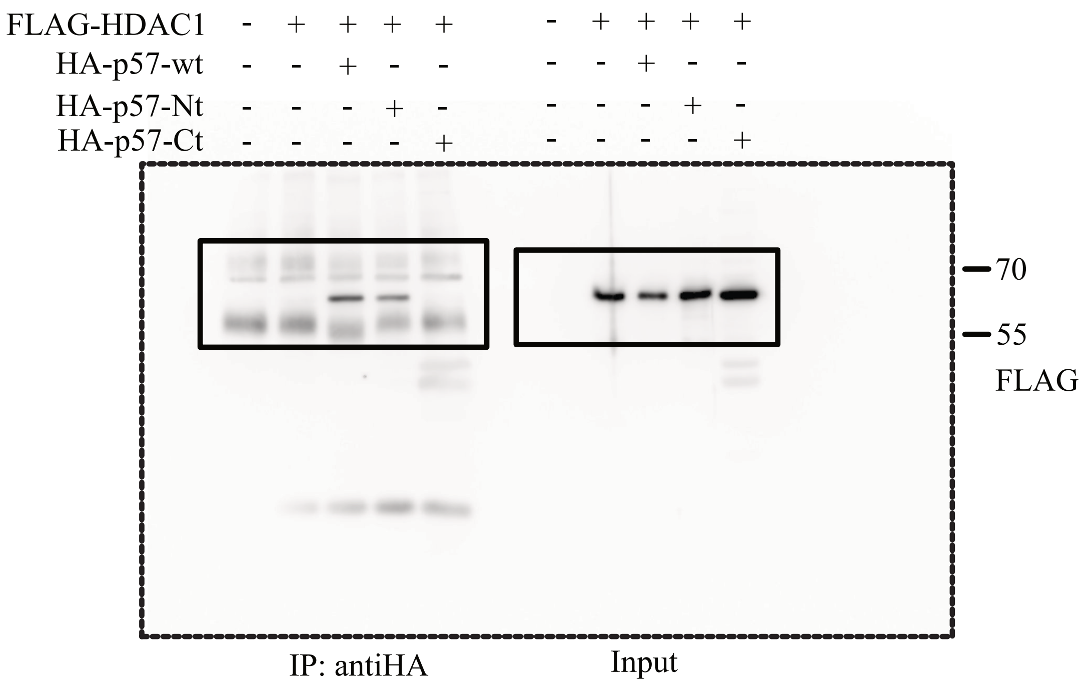

Figure 5A

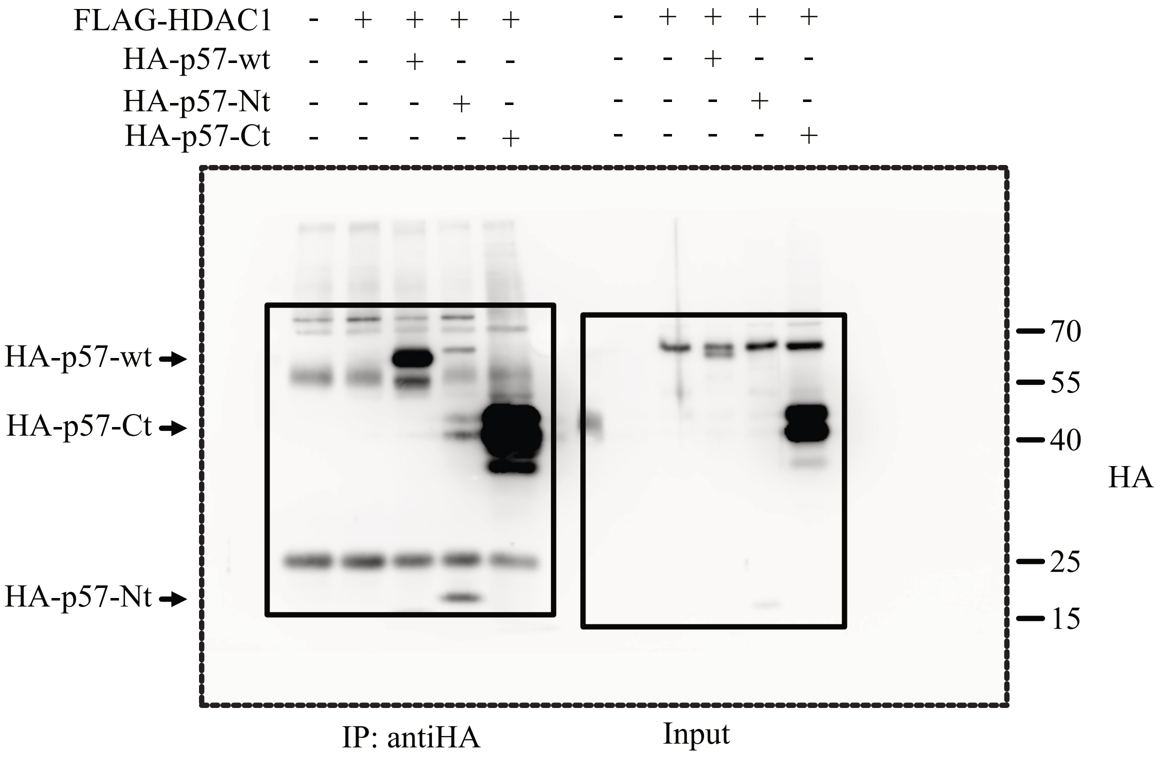

Supplementary figure 7: Original LAS4000 scans cropped for preparing final figure 5B.
Cropped areas and boundaries of Las4000 scans are indicated

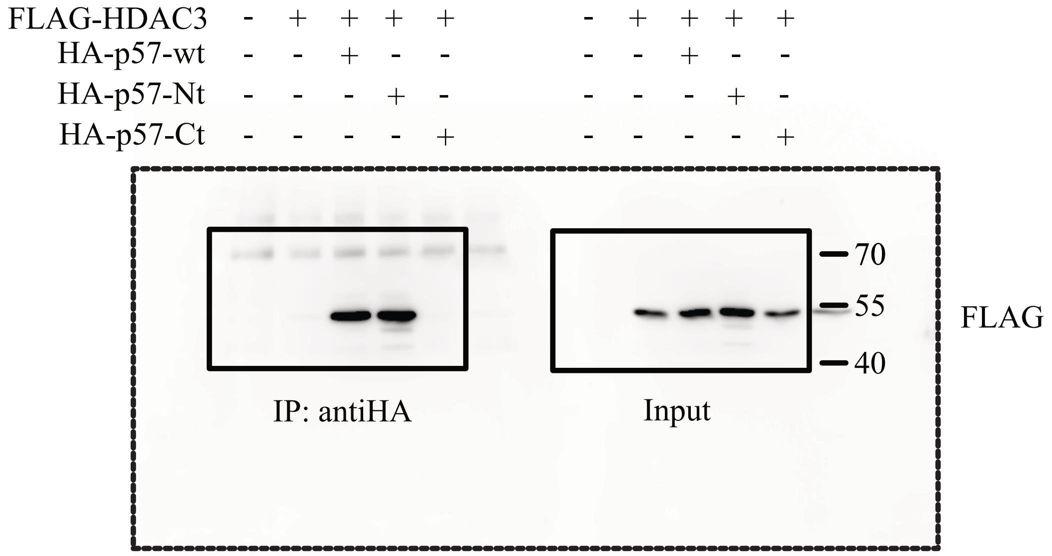

Figure 5B

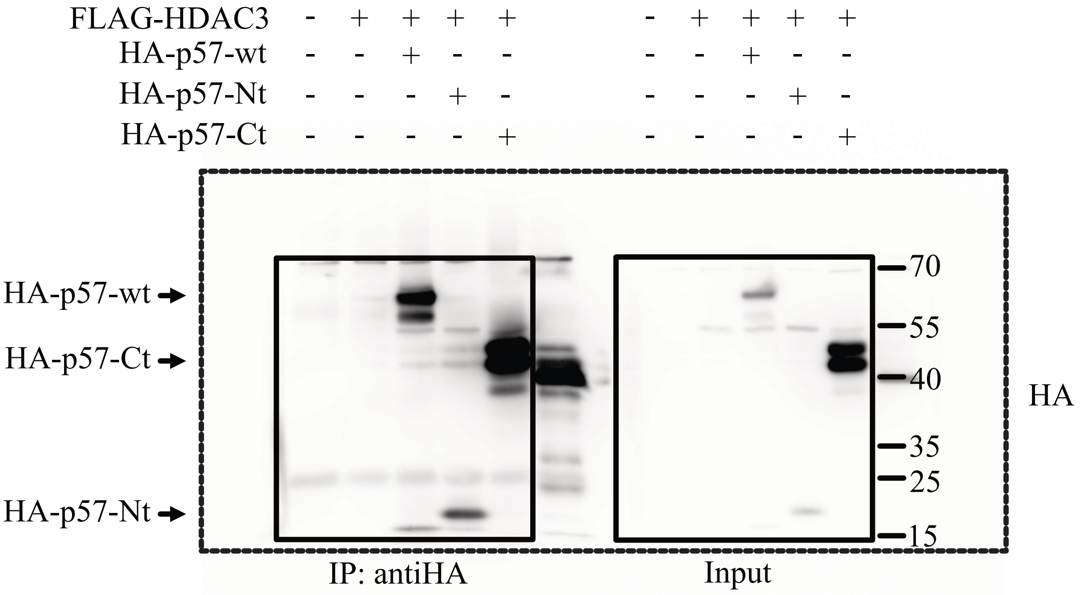

Supplementary figure 8: Original ECL-exposed films cropped for preparing final figure 5C. Cropped areas are indicated.

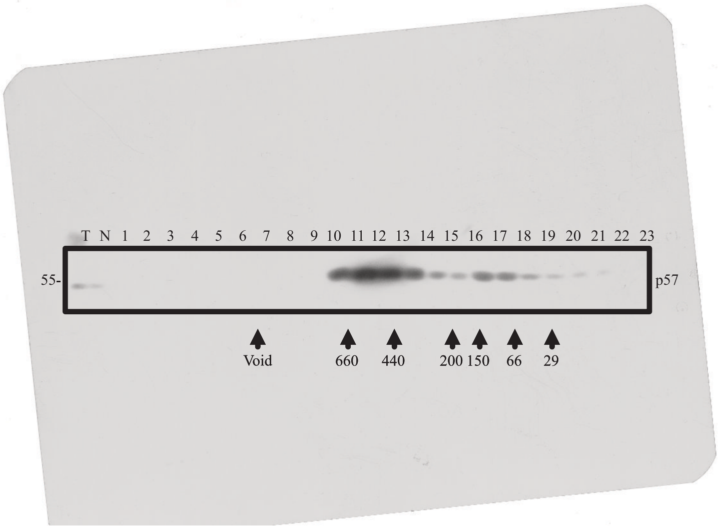

Figure 5C

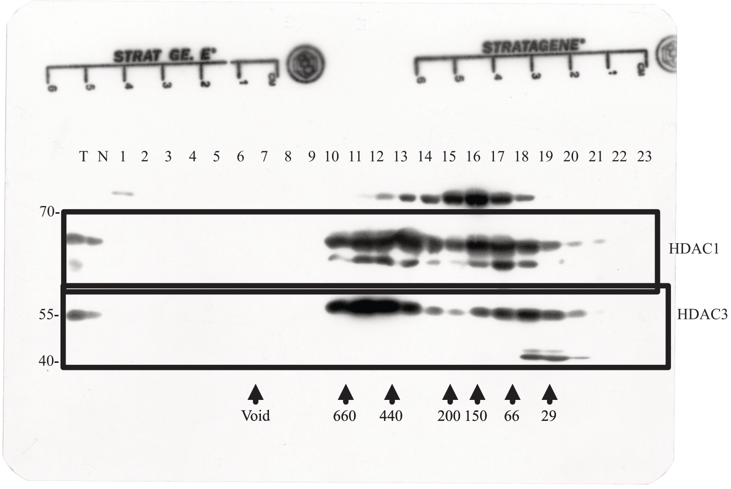

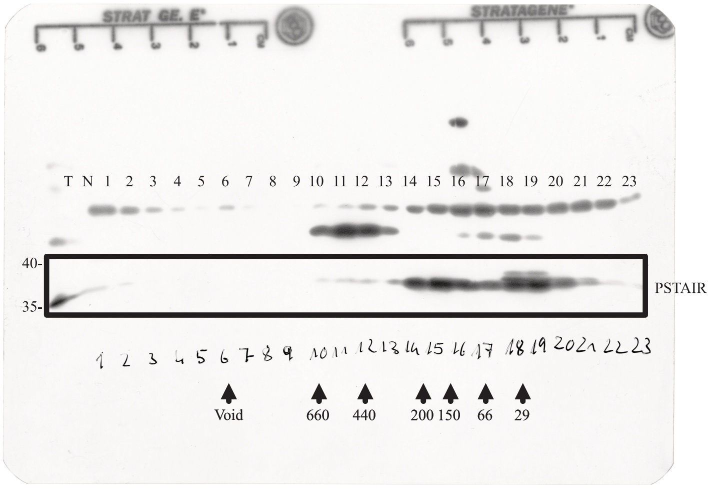

Supplement: Supplementary file 1 [file Data_Sheet_1.PDF]
